# Cellular internalization of bystander nanomaterial induced by TAT-nanoparticles and regulated by extracellular cysteine

**DOI:** 10.1038/s41467-019-11631-w

**Published:** 2019-08-13

**Authors:** Yushuang Wei, Tang Tang, Hong-Bo Pang

**Affiliations:** 1 Department of Pharmaceutics, University of Minnesota, Minneapolis, MN USA; 2 Cancer Center, Sanford Burnham Prebys Medical Discovery Institute, La Jolla, CA USA

**Keywords:** Nanoparticles, Cell delivery, Endocytosis

## Abstract

Entry into cells is necessary for many nanomaterial applications, and a common solution is to functionalize nanoparticles (NPs) with cell-penetrating ligands. Despite intensive studies on these functionalized NPs, little is known about their effect on cellular activities to engulf other cargo from the nearby environment. Here, we use NPs functionalized with TAT (transactivator of transcription) peptide (T-NPs) as an example to investigate their impact on cellular uptake of bystander cargo. We find that T-NP internalization enables cellular uptake of bystander NPs, but not common fluid markers, through a receptor-dependent macropinocytosis pathway. Moreover, the activity of this bystander uptake is stimulated by cysteine presence in the surrounding solution. The cargo selectivity and cysteine regulation are further demonstrated ex vivo and in vivo. These findings reveal another mechanism for NP entry into cells and open up an avenue of studying the interplay among endocytosis, amino acids, and nanomaterial delivery.

## Abstract (web summary)

To enter the cells, nanomaterials often need covalent conjugation with cell-penetrating ligands such as TAT. Here, the authors show that simple mixing with TAT-coupled nanoparticles enables the cellular uptake of unfunctionalized nanoparticles, and its activity is stimulated by cysteine in the medium.

## Introduction

Entry into target cells is a necessary and yet challenging step for many nanomaterial applications^1^. Cell membrane presents a barrier to macromolecules, such as nanoparticles (NPs), to reach their sites of action inside the cells^2^. A common solution is to link NPs with cell-penetrating ligands, such as peptides, aptamers, antibodies, and small molecules (functionalized NPs)^3^. By binding to their receptors on the cell surface, these ligands invoke endocytosis processes that engulf themselves and the coupled NPs. Over the years, a wide variety of such ligands have been described to facilitate the cell entry of NPs^4,5^. The endocytic route and molecular machineries for internalizing these functionalized NPs have also been well documented^2,6–8^. However, one important question has long been overlooked: does the internalization of functionalized NPs influence the cellular activity of engulfing other cargo from the nearby environment?

To simplify the study, we focus here on the effect of functionalized NPs on the cellular uptake of bystander cargo, which are unable to enter cells by themselves and have no physical interaction with functionalized NPs. The uptake of bystander cargo (bystander uptake) occurs naturally in cells through a specialized endocytic pathway, macropinocytosis (MP). This pathway allows cells to non-selectively engulf nearby fluids and substances, which can be traced by fluid markers like dextran and albumin^9,10^. Moreover, MP is thought to occur spontaneously independent of ligand-receptor interactions^11,12^. Thus far, ligand-initiated bystander uptake was only seen with a few cationic cell-penetrating peptides (CPPs)^13^. Transactivator of transcription (TAT) peptide is one of the most used CPPs, and one study showed that it can enhance the bystander uptake of dextran through the MP pathway^14^. Another example arises from the study of CendR peptides, which contain a R/KXXR/K motif on their C-terminus (*C*-end *R*ule, CendR)^4,15^. A number of tumor-specific CendR peptides can induce the transport of bystander cargo, spanning from small molecules to NPs, across the vasculatures and into the tumor tissue and cells in vivo^4,16,17^. However, the exact mechanism and the regulation of the bystander activity are far from understood. To our best knowledge, there is no report on whether these CPPs can still induce such bystander uptake when covalently coupled to NPs.

We previously characterized the endocytic route and regulatory mechanism for cationic CPP-mediated NP entry into cells^18,19^. Our studies showed that both TAT- and CendR-functionalized NPs are engulfed in macropinosome-like structures. However, this process differs from traditional MP in that it depends on CPP-receptor interaction (receptor-dependent MP). More interestingly, its activity was regulated by the concentration of amino acids surrounding the cells, which is also distinct from traditional MP^18^. Here, we use TAT-coupled NPs (T-NPs) as a model system to investigate whether functionalized NPs influence the cellular uptake of bystander cargo. Our results show that T-NP co-administration stimulates the cellular uptake of other NPs in a bystander manner. This bystander activity applies to NPs but not fluid markers, and is regulated by the concentration of cysteine (and its derivatives), but not all other amino acids, in the culture medium.

## Results

### T-NPs stimulate the cellular uptake of bystander NPs

NPs used in this study are summarized in Table 1. When coupled to macromolecules, TAT peptide was reported to bind with heparan sulfate proteoglycans (HSPGs) on the cell surface and induce endocytosis^20,21^. We previously showed that TAT made with *L*-amino acids (*L*-TAT) also binds with the receptor of CendR peptides, neuropilin-1 (NRP1)^19^. To avoid the interference, we used *D*-TAT-NH_2_ (made of *D*-amino acids and thus does not bind with NRP1) to make T-NPs in this study^19^. We first validated the cell entry efficiency of T-NPs. All T-NPs (T-Ag, T-Au, T-IONP and T-QD) were internalized into cells efficiently while NPs alone were not (Supplementary Fig. 1). In this study, we primarily used silver nanoparticles (AgNPs) due to two reasons. First, AgNPs can greatly enhance the fluorescence intensity of the coupled dyes, making it easier to visualize and quantify the NPs internalization (Supplementary Fig. 2). Moreover, an etching technique was developed to rapidly dissolve the silver core and eliminate the fluorescence signals of extracellular AgNPs, while it keeps those already internalized unharmed. This method allows us to accurately monitor and quantify the internalized AgNPs^18,22^. Cellular studies showed that CHO and H1975 cells have the highest activity of engulfing T-Ag (Supplementary Fig. 1 and Supplementary Table 1), which were then used as the primary cell lines below.

**Table 1:** Nanoparticles used in this study

Nanoparticles	Abbreviations	Surface coating	*Z*-size (nm)	Zeta potential (mV)
CF488-AgNP-NAB	Ag-488	CF488A, PEG 1000, NAB	42.51 ± 1.25	−3.6 ± 1.31
CF555-AgNP-NAB	Ag-555	CF555, PEG 1000, NAB	45.41 ± 2.97	−7.05 ± 0.41
CF647-AgNP-NAB	Ag-647	CF647, PEG 1000, NAB	50.26 ± 2.92	−8.54 ± 0.43
CF555-AgNP-*D*-TAT-NH_2_	T-Ag	CF555, PEG 1000, NA-biotin-D-TAT-NH_2_	55.57 ± 3.31	−5.18 ± 0.55
CF647-AuNP-NAB	Au-647	CF647, PEG 1000, NAB	39.42 ± 3.21	−4.93 ± 0.68
CF488-AuNP-*D*-TAT-NH_2_	T-Au-488	CF488A, PEG 1000, NA-biotin-D-TAT-NH_2_	38.13 ± 1.58	−4.26 ± 0.51
CF647-AuNP-*D*-TAT-NH_2_	T-Au	CF647, PEG 1000, NA-biotin-D-TAT-NH_2_	38.68 ± 3.11	−3.49 ± 0.31
AuNP-PEG 50 nm	Au50	PEG 5000	70.72 ± 4.72	−2.41 ± 0.18
CF647-IONP	IONP-647	CF647, Dextran	34.21 ± 0.71	−5.35 ± 1.78
CF647-IONP-*D*-TAT-NH_2_	T-IONP	CF647, Dextran, NA-biotin-D-TAT-NH_2_	36.06 ± 4.43	−2.45 ± 3.18
CF488-QD	QD-488	CF488A, PEG 2000	18.53 ± 0.90	−8.73 ± 0.42
CF647-QD	QD-647	CF647, PEG 2000	19.40 ± 1.40	−6.32 ± 0.28
CF647-QD-*D*-TAT-NH_2_	T-QD	CF647, PEG 2000, NA-biotin-D-TAT-NH_2_	23.70 ± 1.28	−4.18 ± 1.01

Data presented here are mean ± standard deviation (s.d.) of three independent experiments (*n*  = 3). Zeta potential was measured at pH 7.4 for all NPs. Source data are provided as a Source Data file

*NAB* neutravidin (NA) on nanoparticles bind with biotin, *AgNPs* silver nanoparticles, *AuNPs* gold nanoparticles, *IONPs* iron oxide nanoparticles, *QDs* quantum dots

**Fig. 1:**
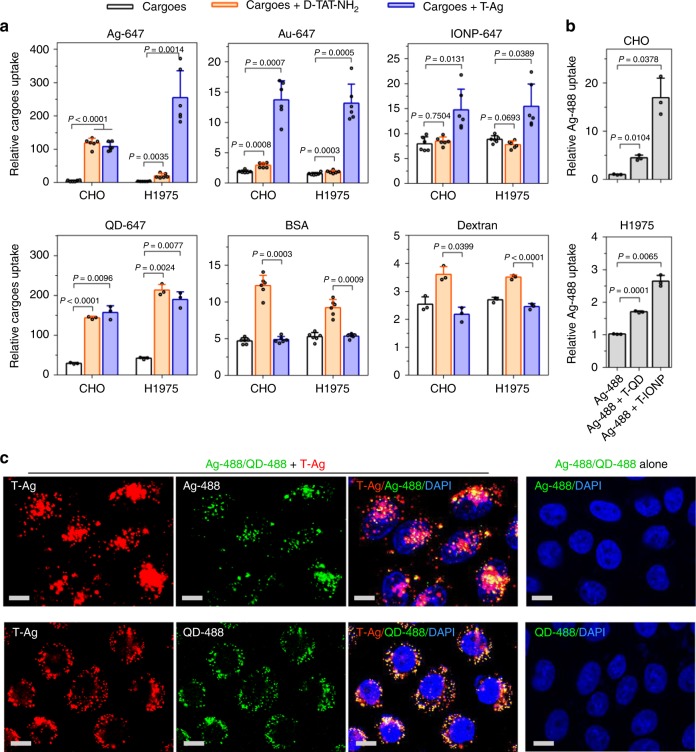
Bystander uptake induced by T-NPs. **a** T-Ag or D-TAT-NH_2_ peptide was mixed with the indicated bystander cargo for internalization in CHO and H1975 cells. After etching or washing, the fluorescence intensity of internalized bystander cargo per cell was quantified using flow cytometry and normalized to that of cells alone (*n* = 6 for Ag-647, Au-647, IONP-647 and BSA; *n* = 3 for QD-647 and dextran). **b** The bystander activity of T-QD and T-IONP to Ag-488 in the indicated cell lines. The fluorescence intensity per cell of internalized bystander Ag-488 was normalized to that of cells incubated with Ag-488 alone (*n* = 3). All quantified data were analyzed using one-way analysis of variance (ANOVA) with Tukey’s multiple comparisons test and are expressed as mean ± s.d. One-way ANOVA, **a** CHO (Ag-647), *F* = 157.63, *P* < 0.0001; H1975 (Ag-647), *F* = 55.31, *P* = 0.0007; CHO (Au-647), *F* = 68.46, *P* = 0.0004; H1975 (Au-647), *F* = 90.38, *P* = 0.0002; CHO (IONP-647), *F* = 14.884, *P* = 0.0065; H1975 (IONP-647), *F* = 13.86, *P* = 0.0120; CHO (QD-647), *F* = 144.8, *P* = 0.0067; H1975 (QD-647), *F* = 291.76, *P* = 0.0004; CHO (BSA), *F* = 126.66, *P* < 0.0001; H1975 (BSA), *F* = 68.50, *P* < 0.0001; CHO (dextran), *F* = 40.29, *P* = 0.0229; H1975 (dextran), *F* = 177.52, *P* = 0.0047. **b** CHO, *F* = 35.72, *P* = 0.0262; H1975, *F* = 184.47, *P* = 0.0049. **c** Confocal images of CHO cells after incubation with T-Ag and the indicated bystander NPs. Three independent experiments (*n* = 3) were performed and representative images are shown here for Ag-488 (upper) or QD-488 (bottom). Scale bars, 10 µm. Source data are provided as a Source Data file

Next, we investigated the bystander activity of T-NPs. Before incubating with cells, T-Ag were mixed with one of various types of bystander cargo, including NPs (AgNPs, AuNPs, IONPs, and QDs) and fluid markers (bovine serum albumin (BSA) and 70 kDa dextran). Though unable to enter the cells themselves, all bystander NPs were internalized efficiently when co-incubated with T-Ag (Fig. 1a). Surprisingly, T-Ag had no or little effect on cellular uptake of fluid markers (Fig. 1a). On the other hand, monomeric TAT peptide enhanced the cellular uptake of fluid markers agreeing with previous report^14^, while its bystander activity only applied to some NP types (AgNPs for CHO cells and QDs for both H1975 and CHO cells) but not all (Fig. 1a). These results show that T-NPs can induce bystander uptake, and they have the selectivity towards NP types of bystander cargo. This study also highlights the difference between monomeric CPPs and those displayed on NPs in multivalence when invoking cellular internalization.

Besides T-Ag, we also tested other types of T-NPs. Both T-QD and T-IONP were able to stimulate the cellular uptake of bystander AgNPs (Fig. 1b). This phenomenon was also observed in a wide variety of cell lines (Supplementary Fig. 3a, b), while the activity of bystander uptake varies depending on the composition of T-NPs and cell types (Fig. 1a, b, Supplementary Fig. 3a). T-Ag uptake was not significantly affected by co-incubation with bystander NPs (Supplementary Fig. 3c). Increasing the concentration of either T-NPs or bystander NPs enhanced the bystander uptake (Supplementary Fig. 4). Inside the cells, the majority of bystander NPs colocalized with T-NPs (Fig. 1c). Overall, these results demonstrate the generality of this T-NP-induced bystander uptake.

There are two requirements for NPs to be qualified as bystander cargo: (i) they are unable to acquire the TAT peptide from T-NPs; (ii) they are not physically attached to T-NPs. We then set out to rule out the possibility that the bystander uptake we observed is due to the violation of the above requirements. We used different surface coating to ensure that some of bystander NPs (AuNPs 50 nm, IONPs, and QDs) have no neutravidin (NA), which was used on T-NPs to conjugate with biotin-TAT peptide. For bystander NPs having NA, we used free biotin to block the binding sites of NA (NAB, refer to Table 1) before mixing with T-NPs. To verify the blocking effectiveness, we mixed free biotin-TAT peptide with NAB-coated bystander NPs. These TAT peptides did not increase the bystander uptake to the level of TAT-coupled NPs, indicating that no or very few biotin-TAT can replace free biotin (Supplementary Fig. 5). Collectively, these results suggest that the bystander uptake we observed is not due to the transfer of TAT peptide between NPs. To rule out the second possibility, bystander NPs (AgNPs, AuNPs, IONPs, and QDs) were incubated with T-Ag on heparan sulfate (HS)-coated plate. After washing to remove unbound NPs, T-Ag, but not any bystander NPs, was found to bind with HS on the plate (Supplementary Fig. 6). To provide additional confidence, we performed the following studies. First, as QDs have different density from other metal NPs, we used centrifugation to separate bystander QDs and T-Ag after incubated in the conditioned medium. We found no QDs precipitated together with T-Ag, indicating that there is no physical interaction between these two NPs (Supplementary Fig. 7a–b). Second, we mixed Au-647 and T-Ag in the cell culture medium before imaging with transmission electron microscopy (TEM). All NPs were scattered under TEM without obvious clustering, indicating that these two NPs do not bind with each other (Supplementary Fig. 7c). All these results, together with the fact that all NPs are coated with hydrophilic polymer and negatively charged, support that there is no physical interaction between T-NPs and their bystander NPs.

### Bystander uptake uses a receptor-dependent MP pathway

**Fig. 2:**
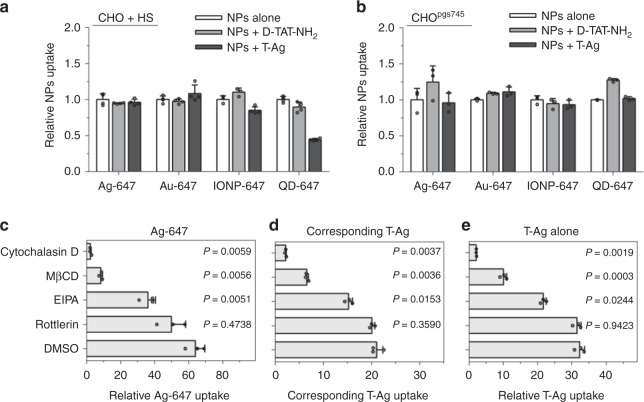
Mechanistic studies of bystander uptake. **a** Bystander uptake is completely abolished by HS. CHO cells were incubated with HS prior to the addition of the indicated bystander NPs (*x*-axis) alone, or with D-TAT-NH_2_ or T-Ag. The fluorescence intensity of bystander NPs per cell was quantified by flow cytometry and normalized to that of bystander NPs alone (*y*-axis). Data presented here are mean ± s.d. of three independent experiments (*n* = 3). **b** The indicated bystander NPs (*x*-axis) were incubated with CHO^pgs745^ cells with D-TAT-NH_2_ or T-Ag, and bystander NPs uptake (*y*-axis) was quantified as described in **a**. Data shown are mean ± s.d. of three independent experiments (*n* = 3). **c**–**e** CHO cells were pre-treated with indicated MP inhibitors dissolved in DMSO (y-axis) before incubating with T-Ag and Ag-647. The fluorescence intensity of AgNPs was quantified by flow cytometry and normalized to that of Ag-647 (for Ag-647 bystander uptake) or Ag-555 (for T-Ag uptake) alone (*x*-axis). **c** bystander uptake of Ag-647; **d** corresponding T-Ag uptake in **c**; **e** the uptake of T-Ag alone. All quantified data were analyzed using one-way ANOVA with Tukey’s multiple comparisons test comparing DMSO versus other MP inhibitors and are expressed as mean ± s.d. of three independent experiments (*n* = 3). One-way ANOVA, **c** *F* = 91.712, *P* = 0.0085; **d** *F* = 829.55, *P* = 0.0001; ***e*** *F* = 641.67, *P* < 0.0001. Source data are provided as a Source Data file

To understand the mechanism of T-NP-induced bystander uptake, we first tested whether it is a receptor-dependent process. Both CHO and H1975 cells expressed HSPGs on the surface (Supplementary Fig. 8). Blocking the TAT-HSPG interaction by free HS completely abolished the ability of T-NPs to either enter the cells themselves or stimulate the bystander uptake (Fig. 2a; Supplementary Figs. 9 and 10). Similar result was observed when incubating T-NPs and bystander NPs with CHO^pgs745^, a CHO mutant cell line that expresses no HSPG^19^ (Fig. 2b; Supplementary Figs. 9 and 10). These results indicate that T-NP-induced bystander uptake requires TAT-HSPG interaction. Considering all NPs used in the study are negatively charged, we also tested the involvement of scavenger receptors (SRs), which have been shown to mediate the cellular uptake of negatively charged molecules and NPs^23–26^. We observed no uptake of T-Ag and bystander AgNPs in cells treated with a SR inhibitor, polynosinic acid (poly I)^23^ (Supplementary Fig. 11). In contrast, the treatment with a negative control compound, polycytidylic acid (poly C)^23^, exhibited no effect on NP internalization (Supplementary Fig. 11). Without TAT coating, NPs failed to enter the cells even in presence of these receptors (Fig. 1 and Supplementary Fig. 1). Together, T-NP-induced bystander uptake relies on T-NP interaction with cell surface receptors.

Next, we investigated the endocytic pathway that T-NPs invoked to engulf bystander NPs. Cells were pre-treated with various endocytic inhibitors, and their effects on the cellular uptake of T-NPs and bystander NPs were quantified. Cytochalasin D, an inhibitor of F-actin, and EIPA, an inhibitor of the Na+/H+ exchange, have been reported to inhibit MP activity^14,27^. The treatment with both inhibitors significantly reduced the uptake of T-Ag as well as bystander AgNPs (Fig. 2c–e; Supplementary Fig. 9c–e). Similar result was observed with MβCD, which disrupts lipid rafts by removing cholesterol from cellular membrane. On the other hand, rottlerin, an inhibitor of fluid phase endocytosis by interaction with protein kinase C^28,29^, exhibited a much milder effect (Fig. 2c–e; Supplementary Fig. 9c–e). These results suggest that T-NP-induced bystander uptake utilizes a lipid raft and/or cholesterol-dependent macropinocytosis machinery^30^. Meanwhile, the responses to inhibitor treatment were quite similar between T-NPs and bystander NPs, indicating that they may be internalized together.

**Fig. 3:**
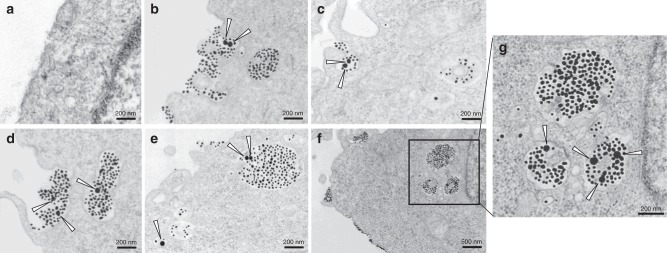
TEM analysis of endocytic structures for bystander uptake. CHO cells were incubated with Au50 alone (**a**) or Au50 + T-Au (**b**–**g**) in DMEM medium for 1 h before being fixed and processed for TEM imaging. The triangle arrows highlight the Au50 particles in the corresponding images. The Au50 particles were found together with T-Au in macropinosome-like endocytic vacuoles (>200-nm in diameter) (**b**–**d**), early endosome (**e**, bottom l**e**ft), and late endosomes and lysosomes (**e**, **g**). Scale bars, 200 nm (except in **f**, in which is 500 nm). Three independent experiments (*n* = 3) were performed and representative TEM images are shown here. Source data are provided as a Source Data file

TEM was then used to directly visualize the endocytic structure and subcellular transport route for bystander NPs at the ultrastructural level. Gold nanoparticles (50-nm in diameter, Au50), which were used as bystander NPs, did not enter the cells by themselves (Fig. 3a). When mixed with T-Au (~20 nm), Au50 were internalized together with T-Au in the same macropinosome-like (>200 nm in diameter) vacuoles (Fig. 3b–d). Inside the cells, Au50 and T-Au were colocalized in some, but not all, endocytic vesicles. T-Au and Au50 appeared sequentially in early endosomes, late endosomes/multivesicular bodies, and eventually lysosomes (Fig. 3b–g). Together, the above results support the notion that a receptor-dependent MP process initiated by T-NPs can engulf bystander NPs into the same endocytic structures, and transport them through endosomal compartments inside the cells. Furthermore, TEM studies show that T-Au, another T-NP type, can induce the bystander uptake. There is also no obvious aggregate between these two AuNPs, further proving that the bystander uptake is not due to physical interactions between NPs.

### Cysteine stimulates T-NP-induced bystander uptake

**Fig. 4:**
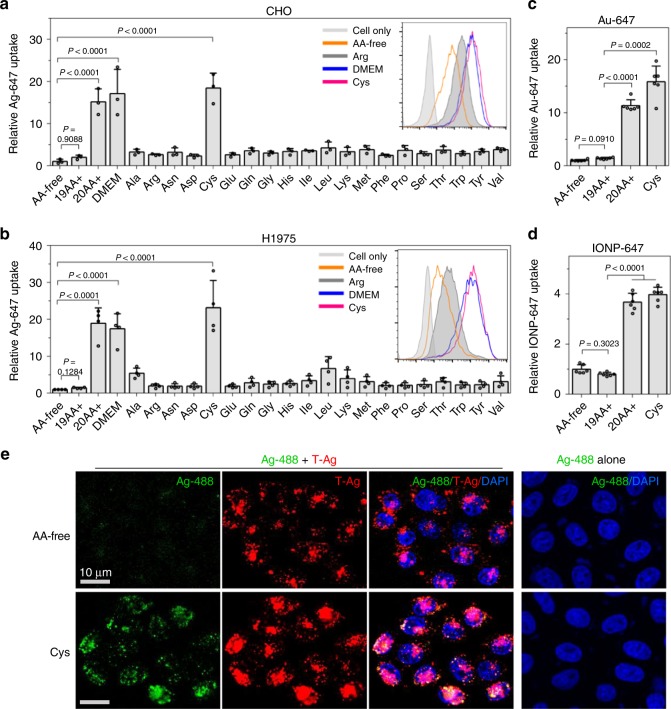
Extracellular Cysteine stimulates the T-NP-induced bystander uptake. **a**, **b** Bystander uptake of Ag-647 in cells cultured in media containing different amino acid compositions. CHO (**a**) and H1975 (**b**) cells were incubated with Ag-647 and T-Ag in AA-free, 19AA+, 20AA+, DMEM or individual AA medium. After etching and washing, the fluorescence intensity per cell of internalized Ag-647 was determined by flow cytometry, and normalized to that in AA-free medium. Data are expressed as mean ± s.d. (*n* = 3 in **a**, *n* = 4 in **b**). The insertion in **a**, **b** are the representative flow cytometry histograms. **c**, **d** CHO cells were incubated with T-Ag and Au-647 (**c**) or IONP-647 (**d**) in AA-free, 19AA+, 20AA+, and Cys medium. The internalized NPs as fluorescence intensity per cell was normalized to that in AA-free medium (*n* = 6). All quantified data **a**–**d** were analyzed using one-way ANOVA with Tukey’s multiple comparisons test and are presented as mean ± s.d. One-way ANOVA, **a** *F* = 35.772, *P* = 0.0050; **b** *F* = 45.256, *P* = 0.0004; **c** *F* = 24.358, *P* = 0.0183; **d** F = 102.60, *P* = 0.0016. **e** Confocal microscope images of CHO cells after incubation with T-Ag and Ag-488 in AA-free (upper) and Cys (bottom) medium. Three independent experiments (*n* = 3) were performed and representative images are shown here. Scale bars, 10 µm. Source data are provided as a Source Data file

We recently discovered that the concentration of extracellular amino acids (AAs), through mTOR signaling, regulates the efficiency of CendR-mediated NPs internalization^18^. Here, the effect of AAs was investigated on the uptake of T-NPs and their bystander cargo. Special cell culture medium was prepared according to DMEM formulation without any amino acid (AA-free), or supplemented with: all 20 amino acids (20AA+), 19 amino acids without cysteine (19AA+), or only the indicated amino acid (e.g. Ala, Cys, Leu, etc) (Supplementary Tables 2 and 3). All media contained fetal bovine serum (FBS) that was pre-dialyzed to eliminate free AAs. When cultured in AA-free medium, cells exhibited a lower activity to engulf T-Ag than 20AA+ or commercial DMEM medium (Supplementary Fig. 12). The addition of only cysteine (Cys) to AA-free medium was able to recover the T-Ag uptake to the level of 20AA+ medium (Supplementary Fig. 12). A much more striking effect was seen with T-NP-induced bystander uptake. Compared to AA-free medium, both 20AA+ and Cys medium significantly increased the cellular uptake of bystander NPs by 15-fold or more when co-incubated with T-Ag (Fig. 4a, b). In contrast, 19AA+ medium or medium containing individual amino acid other than Cys behaved similarly to AA-free medium. This Cys regulation was validated in eight other cell lines (Supplementary Fig. 13a, b). The difference of bystander uptake in AA-free and Cys medium was also proven by confocal images (Fig. 4e).

The Cys regulation was also found when other NP types were used as bystander cargo. Cells internalized more Au-647 or IONP-647 when incubated with T-Ag in Cys and 20AA+ medium than those in AA-free and 19AA+ medium (Fig. 4c, d; Supplementary Fig. 13c, d). However, bystander uptake of QDs in Cys and 20AA+ medium was lower than that in AA-free and 19AA+ medium (Supplementary Fig. 13e, f). Without T-NPs, there was no difference on the uptake of bystander NPs in AA-free or media with Cys (Supplementary Fig. 13g–j), suggesting that Cys regulation depends on ligand-receptor interaction. Overall, these results demonstrate a role of extracellular Cys in regulating the cellular uptake of T-NPs, and more evidently, their bystander activity.

### Characterization of Cys regulation on bystander uptake

**Fig. 5:**
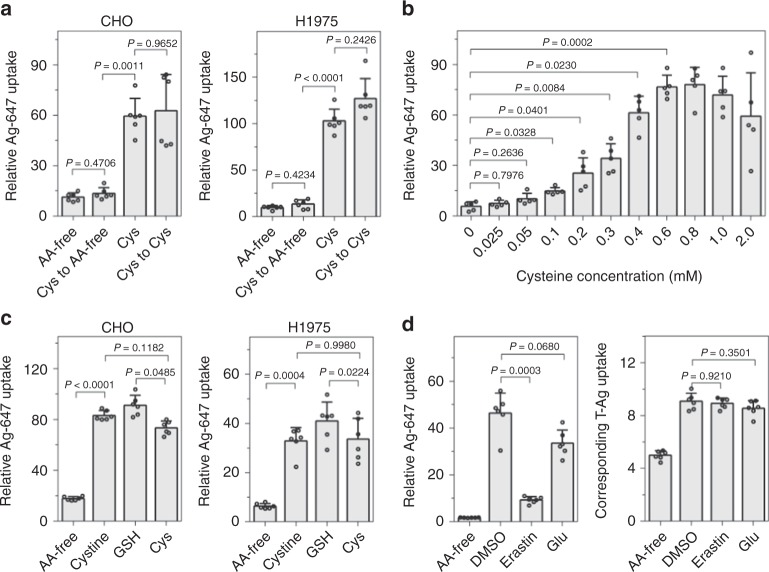
Characterization of Cys regulation of bystander uptake. **a** Cysteine presence is necessary for the increase of Ag-647 bystander uptake. CHO (left) and H1975 (right) cells were pre-incubated in Cys medium before exposed to Ag-647 and T-Ag in AA-free (Cys to AA-) or Cys medium (Cys to Cys). **b** The bystander uptake of Ag-647 was increased as Cys concentration increased. CHO cells were incubated with Ag-647 and T-Ag in AA-free medium with gradually increasing concentrations of Cys from 0 to 2 mM. **c** T-Ag-induced bystander uptake is stimulated by cystine and GSH. CHO (left) and H1975 (right) cells were incubated in cystine or GSH containing medium with Ag-647 and T-Ag. **d** Bystander uptake of Ag-647 was inhibited by cystine inhibitors. CHO cells were pre-treated with erastin or glutamate for 10 min prior to the incubation with Ag-647 and T-Ag for 30 min in the presence of erastin or glutamate. Left, bystander uptake of Ag-647; Right, uptake of corresponding T-Ag. For all above panels, the cells were etched after incubation, and the fluorescence intensity per cell of internalized NPs was determined by flow cytometry, and normalized to that of Ag-647 (For Ag-647 bystander uptake in **a**–**d** (left)) or Ag-555 (For T-Ag uptake in **d**(right)) alone. All quantified data **a**–**d** were analyzed using one-way ANOVA with Tukey’s multiple comparisons and are presented as mean ± s.d. (*n* = 6 for **a**, **c**, and **d**; *n* = 5 for **b**). One-way ANOVA, **a** (left), *F* = 40.268, *P* < 0.0001; **a** (right), *F* **=** 160.20, *P* < 0.0001; **b**, *F* = 62.055, *P* < 0.0001; **c** (left), *F* = 110.33, *P* < 0.0001; **c** (right), *F* = 91.663, *P* < 0.0001; **d** (left), *F* = 155.91, *P* < 0.0001; **d** (right), *F* = 61.985, *P* = 0.0001. Source data are provided as a Source Data file

The Cys regulation occurred within 1-h incubation time and required no pre-incubation (Fig. 5a; Supplementary Fig. 14a). HSPG staining also revealed that culture in Cys medium has little effect on the expression level of HSPG on the cell surface (Supplementary Fig. 8). These results indicate that Cys regulation is a spontaneous cellular response, rather than through a long-term effect such as the overexpression of surface HSPG. To further understand the Cys regulation, we first examined whether the Cys presence was necessary for its regulation on T-NP bystander uptake. Cells were pre-incubated in Cys medium before being changed to fresh AA-free medium containing T-Ag and bystander NPs. Pre-exposure to Cys did not stimulate the bystander uptake as observed in cells incubated in Cys medium, suggesting the presence of Cys is required for its stimulatory effect (Fig. 5a; Supplementary Fig. 14). Next, cells were incubated in media with gradually increasing concentrations of Cys from 0 to 2 mM. The uptake of bystander NPs gradually augmented with increasing Cys concentration before reaching a plateau (0.4 mM for CHO cells, 0.1 mM for H1975 cells) (Fig. 5b; Supplementary Fig 14b). This result suggests that the Cys regulation on bystander uptake is concentration-dependent, and the sensitivity to Cys varies with cell types. Lastly, it is well known that the sulfhydryl group of Cys has a high affinity for heavy metals^31^. To examine if Cys functions through modifying the metal NPs, T-Ag and Ag-647 were pre-incubated in Cys medium before change to AA-free or Cys medium for incubation with cells. Pre-incubation of NPs with Cys had little effect on T-NP-induced bystander uptake in the AA-free medium (Supplementary Fig. 15). Besides, the size and electrostatic charge of NPs did not change when incubated with Cys (Table 1 and Supplementary Table 4). Therefore, Cys regulation is a cellular response rather than through affecting T-NPs or bystander NPs.

Cys is often oxidized to generate the derivative cystine (CysS–SCys). In mammals, cystine, but not Cys, is abundant in plasma and extracellular fluid^32^. Cystine is also taken up by cells for synthesizing the antioxidant, glutathione (GSH)^33,34^. GSH is present inside cells in millimolar concentration to protect from oxidative stress^35^. We tested whether these Cys derivatives have similar stimulatory effect on T-NP bystander uptake. Indeed, when added to AA-free medium, both cystine and GSH enhanced the cellular uptake of T-Ag and the bystander NPs similar to Cys (Fig. 5c; Supplementary Fig. 14c).

Besides T-NPs, we also found that Cys can promote the uptake of bystander NPs induced by TAT peptide (Supplementary Fig. 16a). However, this Cys stimulation effect did not apply to dextran (Supplementary Fig. 16b), suggesting that Cys regulation is also specific to NP-type bystander cargo.

Cys and cystine are transported into cells via the anionic AA transport system, system Xc^-^^32^. We utilized inhibitors to investigate whether Cys regulation depends on the transport of Cys or cystine into cells. After pre-incubation with glutamate or erastin (system Xc- inhibitors, which blocks the cellular uptake of Cys and cystine)^36,37^, CHO cells were incubated with T-Ag and AgNPs for internalization. The bystander uptake of AgNPs was significantly inhibited by erastin and glutamate, while these inhibitors had little effect on T-Ag uptake (Fig. 5d). This result indicates that Cys regulation on bystander NPs uptake depends on the transport of Cys or cystine into cells. We also tested these inhibitors on other cell lines. While erastin gave rise to similar result, glutamate showed no inhibition in H1975 cells, which may be due to their different sensitivity to Cys concentration (Supplementary Fig. 14d).

### T-NP-induced bystander uptake under physiological conditions

**Fig. 6:**
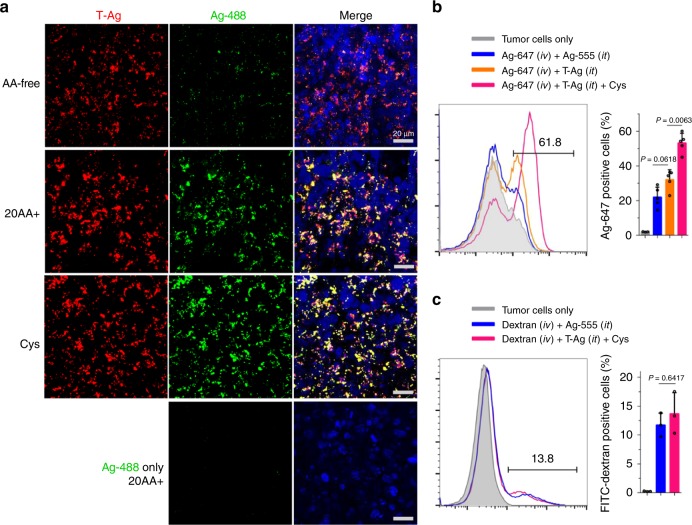
Demonstration of T-NP-induced bystander uptake under physiological conditions. **a** Bystander uptake of Ag-488 by live tumor slices. T-Ag and Ag-488 were incubated with live 4T1 tumor slices in AA-free, 20AA+, and Cys medium for 2 h. After incubation, slices were etched, stained with DAPI (blue) and imaged by confocal microscope (*n* = 3 mice for each group). Scale bars are 20 µm. **b**, **c** The bystander uptake of Ag-647 (**b**) or dextran (**c**) in 4T1 tumors. 4T1 tumors bearing mice were received Intratumoral (i.t.) injection of T-Ag (±Cys) prior to the intravenous (i.v.) injection of Ag-647 (±Cys) or FITC-dextran (±Cys). Two hours after circulation, mice were transcardial perfused with PBS, and then tumors were collected and further dissociated to single cells, etched and quantified by flow cytometry. The amount of Ag-647 or FITC-dextran positive cells in percentage was shown. Data are represented as the mean ± s.d. of at least three mice for each group (**b** *n* = 3 for control and *n* = 5 for experimental groups; *n* = 3 for **c**). Quantified data **b**, **c** were analyzed using one-way ANOVA with Tukey’s multiple comparisons test. One-way ANOVA, **b**, *F* = 60.141, *P* < 0.0001; **c** *F* = 33.373, *P* = 0.0100. Source data are provided as a Source Data file

To investigate whether the bystander uptake phenomenon exists under physiological conditions, we performed ex vivo and in vivo studies using live tumor slices and subcutaneous tumor xenograft as previously described^18^. Without TAT, AgNPs were unable to enter the live tumor slices (Fig. 6a). Instead, T-Ag was effectively internalized by these tumor slices along with bystander AgNPs (Fig. 6a). Compared to AA-free medium, the uptake of bystander AgNPs was significantly increased when slices were cultured in medium containing Cys. Meanwhile, the uptake of related T-Ag was also promoted by Cys but in a much less extent. Under these conditions, bystander AgNPs colocalized with T-Ag, supporting the notion that T-Ag bring their bystander NPs into the slices through the same endocytic vesicles as seen in TEM studies (Fig. 3; Fig. 6a). Similar experiments with dextran showed that neither T-Ag nor Cys presence promoted the tissue uptake of dextran (Supplementary Fig. 17), confirming the cargo selectivity and Cys regulation in the physiological tissues.

To demonstrate the bystander uptake in vivo, we injected bystander cargo (AgNPs or dextran) intravenously, while T-Ag was injected locally into tumors with or without Cys. To eliminate the interference from injection and AgNPs, we used the intratumoral injection of AgNPs (with the same fluorescence dye to T-Ag) as the control to T-Ag. We found that upon intratumoral injection of T-Ag and Cys, the tumor uptake of bystander AgNPs was significantly increased (Fig. 6b). In contrast, dextran uptake by tumor cells was not changed by co-administration of T-Ag and Cys (Fig. 6c). Together, these ex vivo and in vivo studies demonstrate that T-NPs can stimulate the bystander uptake of only NP-type bystander cargo in vivo, and the presence of Cys significantly increases the activity of this bystander uptake.

## Discussion

TAT and other cationic CPPs have been widely used to deliver nanomaterial and other cargo types into cells in vitro and in vivo^14,19,20,38,39^. The common method is to conjugate TAT with NPs, whose cell encounter invokes a receptor-dependent MP process for cell entry^19,20^. Here, we report another mechanism for NP entry into cells aided by T-NPs: when simply co-administered with T-NPs, bystander NPs, without any cell-penetrating ligands, can enter into the cells through the same endocytic pathway as T-NPs. Notably, unlike TAT peptide, T-NPs only induce the bystander uptake of NP-type cargo but not common fluid markers. Moreover, the presence of Cys in the extracellular solution, but not any other amino acids, significantly enhances this T-NP-induced bystander uptake.

Entry into cells in a bystander manner is of several unique advantages to nanomaterial applications. It requires no modification of NPs with cell-penetrating ligands, which might interfere with NPs functions. And it is exempted from the limitation of the number of receptors available at cell surface. In some cases, especially in vivo conditions, the number of receptors can be limited to allow sufficient ligand binding and subsequent internalization^40^. Bystander uptake exists naturally in cells through traditional MP pathway, but it is rather a nonselective and receptor-independent process^8,11^. Our previous studies on the cell entry of T-NPs and CendR-NPs unveiled a receptor-dependent MP pathway. And TAT peptide was previously shown to enhance the bystander uptake of dextran through MP pathway^9,18,19^. These studies intrigued us to investigate whether CPPs, when coupled to NPs, can induce cellular uptake of bystander cargo like NPs. TAT was used here as the model CPP due to its ability to enter a wide variety of cell types. Here, we indeed found that T-NPs were able to stimulate cellular uptake of bystander NPs. This phenomenon was validated with a variety of compositions of T-NPs and bystander NPs, in multiple cell lines, and under physiological conditions. Among the bystander NPs, T-Ag showed the highest bystander uptake effect on AgNPs, followed by AuNPs and QDs, and the lowest on IONPs. On the other hand, T-QD showed much lower bystander activity to AgNPs than T-Ag. The bystander uptake of the same NPs in different cell lines also varies. The exact features of T-NPs and bystander NPs to regulate the bystander activity remain to be investigated.

The endocytic mechanism of bystander uptake was characterized by various methodologies. The uptake of T-NPs and bystander NPs showed similar sensitivity to a panel of endocytic inhibitors, and the majority of bystander NPs was found together with T-NPs when observed with confocal and TEM imaging. The efficiency of bystander uptake was enhanced by a higher concentration of T-NPs or bystander NPs. Thus, our results indicate that T-NPs, by invoking a receptor-dependent MP process, bring bystander NPs into cells in the same endocytic vesicles. We speculate that T-NP-induced macropinosomes provide a “hitch ride” to bystander NPs for cell entry, although we have extensively demonstrated no physical interaction between T-NP and bystander NPs. The exact cell biology underlying this phenomenon remains to be further studies, especially in regard to its unique properties of cargo selectivity and Cys regulation.

The first unanticipated feature of T-NP-induced bystander uptake is the selectivity of bystander cargo. Unlike TAT peptide, T-NP can only increase the cellular uptake of bystander NPs but not fluid markers (dextran or BSA). As previously reported, TAT peptide enters the cells through both direct fusion and multiple forms of endocytosis, and it is not always dependent on cell surface receptors like T-NPs^14,19,38^. Our findings further highlight the functional difference between the cellular processes invoked by free peptide and NP-formulated ones. Multivalent display of TAT peptide on NPs, together with other possible factors (e.g. size of NP carrier), may initiate endocytic processes having distinct cargo uptake properties from free peptide. A direct result may be cargo selectivity. The endocytic structures invoked by T-NP are >200 nm, which may be particularly active to engulf bystander NPs (~20 nm or more in diameter) than dextran and BSA (<10 nm)^41^. The understanding of these phenomena awaits further elucidation of endocytic machineries invoked by free TAT peptide and T-NPs of various features (sizes, core compositions, TAT density, etc.).

Cys regulation is another finding about T-NP-induced bystander uptake. Our previous work found that extracellular amino acid regulates the cell entry activity of CendR-coated NPs^18^. This inspired us to investigate the effect of every individual AA on the uptake of T-NPs and bystander NPs. Surprisingly, we revealed that Cys is the only AA to increase the cellular uptake of not only T-NPs themselves, but also bystander NPs to a much more extent. The Cys stimulation was again confirmed in various compositions of T-NPs, bystander NPs, and various cell lines. T-NPs binding to receptors and Cys presence are required for Cys regulation of bystander uptake, while Cys does not change the sizes and charges of bystander NPs or HSPG expression. These results suggest that Cys regulation is again a feature for receptor-dependent MP induced by T-NPs. This conclusion is further supported by the fact that Cys stimulation has the selectivity towards some of NP cargo but not fluid markers like dextran.

Interestingly, the activity of T-NP-induced bystander uptake is correlated with the concentration of extracellular Cys, suggesting that Cys regulation may relate to the concurrent cellular uptake of Cys. What’s more, we found that cystine and GSH, two abundant derivatives of Cys naturally in the body, also promoted the bystander uptake. The inhibition of Cys and cystine transporters reduced the cellular internalization of bystander NPs. Collectively, the Cys regulation on receptor-dependent MP may arise from the Cys transport machinery. Lastly, T-NP-induced bystander uptake and the Cys regulation were validated with live tissues and in live animals, proving the generality of this phenomenon in vivo.

In summary, we show here that T-NPs, by invoking a receptor-dependent MP pathway, can induce the cellular uptake of bystander NPs. These findings provide a mechanism to synergize NP entry into cells, and by studying the NP entry, we can discover unique features of a specialized endocytic pathway. Moreover, Cys regulation establishes a linkage between individual AAs and endocytic activities. Together, our findings provide insights into the interplay among endocytosis, metabolism, and NP delivery.

## Methods

### Reagents and cell lines

CHO wild-type (CHO) and pgs745 (CHO^pgs745^), 4T1, Hela, PPC1 cell lines were purchased from the American Type Culture Collection (Manassas, VA). H1975, H2122 and A549 cells were obtained from Dr. Garth Powis, Cancer Center, Sanford Burnham Prebys Medical Discovery Institute (SBP). MIA PaCa2 cells were provided by Dr. Commisso (SBP Medical Discovery Institute). KPC cells were received from Dr. Andre Nel, University of California, Los Angeles. LL/2 Red-Fluc cells were purchased from PerkinElmer, Inc. (Waltham, MA). Among these, PPC1 cells are a commonly misidentified cell line listed in ICLAC database. We have routinely used PPC1 as a NRP1-positive cell line^19^, and it has been previously authenticated^42^. H1975, A549, H2122 and MIA PaCa2 are authenticated by Short Tandem Repeat (STR) Analysis. The other cells are not authenticated in this study: CHO, Hela and LL/2 cells are recently acquired, and the origin of the cells is not critical for our purposes because we only used these cells to demonstrate the generality of the results in different cellular context. We have tested all cell lines to rule out mycoplasma contamination. CHO and CHO^pgs745^ cells were cultured in Ham’s F-12K (Kaighn’s) Medium (F-12K, Thermo Scientific); H1975, H2122 and A549 cells were cultured in RPMI 1640 medium (Thermo Scientific); 4T1, Hela, PPC1, MIA PaCa2, KPC and LL/2 cells were cultured in Dulbecco’s Modified Eagle’s medium (DMEM, Cat# SH30022, Hyclone). All media were supplemented with 10% fetal bovine serum (FBS), 1% penicillin/streptomycin and 2 mM L-glutamine.

All fluorescence dyes, CF488A, CF555 and CF647 Succinimidyl Ester were purchased from Biotium. Endocytic probes used here were FITC-BSA (Cat# A23015) and FITC-dextran (MW = 70, 000, cat# D1822) from Invitrogen. D-TAT-NH_2_ peptide and biotin-D-TAT-NH_2_ peptides were purchased from LifeTein, LLC (Somerset, NJ). PEG coated 50 nm AuNPs (Cat# AAJ67036-AMI) were purchased from Alfa Aesar. All amino acids were purchased from Sigma and the related Cat# were listed in supplementary table 3. All MP inhibitors, ethyl-isopropyl amiloride (EIPA, cat# 3378/10), Rottlerin (Cat# 1610/10), cytochalasin D (Cat# BML-T109-001), and methyl-β-cyclodextrin (MβCD, cat# 377110050) were purchased from Fisher Scientific. Scavenger receptor inhibitors, polyinosinic acid (Poly I, cat# P4154) and polycytidylic acid (Poly C, cat# P4903) were purchased from Sigma. Antibodies used were mouse anti-HSPG antibody (Clone F58-10E4, Cat# 370255-1, Seigaku), rat anti-HSPG antibody (Clone SPM255, Cat# V2601SAF, NSJ Bioreagents), Donkey anti-mouse AF488 IgG (Cat# A21202, Invitrogen) and Goat anti-rat AF647 IgG (Cat# A21247, Invitrogen) with 1: 200 dilution.

### Nanoparticles preparation

The detailed protocols for nanoparticle preparation are provided in the Supplementary Methods.

AgNPs and AuNPs: Peptide-coated AgNPs and AuNPs (T-Ag-555 and T-Ag-647; T-Au-647, and T-Au-488) were assembled through biotin-neutravidin interaction. AgNPs carried with CF488A, CF555, and CF647, and AuNPs carried with CF488A and CF647 were blocked with biotin through biotin-neutravidin interaction and served as bystander NPs or control NPs.

IONPs: D-TAT-NH_2_ peptide conjugated, CF647 labeled IONPs were obtained as T-IONP. IONPs labeled with only CF647 (IONP-647) were used as one of bystander NPs.

QDs: The PEG2000-NH_2_ coated QDs were finally synthesized and labeled with CF488 or CF647 (QD-488 or QD-647) as bystander NPs. Similar to IONPs, D-TAT-NH_2_ peptide was conjugated to CF647 labeled QDs to obtain T-QD.

### Dynamic light scatter (DLS)

The hydrodynamic size and zeta-potential of the resulting NPs were determined in PBS with a Nano-ZS90 particle analyzer (Malvern, United Kingdom). To investigate the impact of Cys on NPs, T-Ag, Ag-647, IONP-647, and QD-647 were individually incubated in Cys medium at 37 °C for 1 h, then the size and zeta potential were measured by Nano-ZS90.

### Cellular uptake study

The final concentration of NPs used in this study was as listed below, if not otherwise indicated: AgNPs, 2 μL/100 μL medium, about 0.27 nM; AuNPs, 2 μL/100 μL medium, about 0.79 nM; IONPs, 50 µg Fe/mL; QDs, 50 µg/mL. All cells were seeded onto the flat-bottom 96-well plate (Cat# 2870-1002, Nest Scientific, Inc.) at 1 × 10^4^ cells/well in corresponding growth medium for 48 h before being treated with NPs, if not otherwise indicated. All media used for NPs incubation with cells were supplied with 10% dialyzed FBS if not otherwise indicated. After incubation, etching buffer (a final concentration of 10 mM Sodium thiosulfate pentahydrate (Na_2_S_2_O_3_·5H_2_O, CAS# 10102-17-7, Sigma) and 10 mM Tripotassium hexacyanoferrate (III) (K_3_Fe(III)CN_6_, CAS# 13746-66-2, Sigma) in DPBS (Cat# SH30028.02, Hyclone))^22^ was added into the medium and incubated for 1 min to dissolve extracellular AgNPs before being washed twice with DPBS. Thereafter, cells were detached with 0.25% Trypsin-EDTA (Cat# 25300-054, Gibco) and fixed using 4% paraformaldehyde (PFA, Cat# sc-281692, Santa Cruz Biotech.), then analyzed by flow cytometry on Novocyte 3000 (ACEA Biosciences, Inc. San Diego, CA) and BD LSRFortessa (BD Biosciences, San Jose, CA). This etching–washing–detaching–fixation and flow cytometry analysis procedure was performed similarly for all cell uptake experiments in this study.

To quantify the cellular uptake of T-NPs, the indicated cells were incubated with T-NPs in DMEM medium for 1 h at 37 °C before etching (for AgNPs) or washing (for other NPs) to remove extracellular particles. For bystander uptake, bystander NPs (Ag-647, Au-647, IONP-647 or QD-647) were, respectively, mixed with T-Ag or 10 µM D-TAT-NH_2_ peptide in DMEM medium and then incubated with cells for 1 h at 37 °C. For AgNPs bystander uptake induced by T-NPs other than T-Ag, cells were incubated in DMEM medium mixed with Ag-488 plus T-IONP or T-QD for 1 h. For the bystander uptake of macromolecules, cells were incubated in FBS free DMEM medium for 16~18 h^43^ before the incubation with 0.2 mg/mL FITC-BSA or FITC-dextran in FBS free medium plus T-Ag or 10 µM D-TAT-NH2 peptide for 30 min.

To study the impact of individual amino acid on bystander uptake, cells were incubated in AA-free, 20AA+ or the other 20 kinds of individual AA-containing medium with Ag-647 and T-Ag, or T-Ag alone for 1 h. Cells were also incubated with T-Ag and other bystander NPs (Au-647, IONP-647 or QD-647) in AA-free, 19AA+, 20AA+, and Cys medium to investigate the effect of Cys on the bystander uptake to other types of NPs.

To validate Cys regulation on bystander uptake, cells were incubated in 19AA + medium mixed with Ag-647 and T-Ag for 1 h. To check the necessity of Cys presence for bystander uptake, cells were pre-incubated in AA-free, 20AA+ or Cys medium for 1 h, washed by DPBS twice and then incubated in the corresponding AA-free, 20AA+ or Cys medium mixed with Ag-647 and T-Ag, or all incubated in AA-free medium mixed with Ag-647 and T-Ag for 1 h. To investigate the effect of Cys concentration on bystander uptake, cells were incubated with Ag-647 and T-Ag in medium with gradually increased concentrations of Cys from 0 to 2 mM for 1 h. To explore cystine effect on bystander uptake, cells were incubated with Ag-647 and T-Ag in AA-free medium plus 0.4 mM cystine or 0.4 mM glutathione for 1 h. Cells were also incubated with only T-Ag in these media to investigate the effect of cystine and glutathione on T-Ag uptake.

### Flow cytometry

All cells were suspended and fixed in 4% PFA as described above and then analyzed on a Novocyte 3000 Flow Cytometer and BD LSRFortessa (BD Biosciences, San Jose, CA). The median value of corresponding fluorescence intensity of all gated live single cells was used to evaluate the internalization of related NPs or macromolecules.

### Physical interaction study

T-Ag was mixed with Ag-647, Au-647, IONP-647 or QD-647 in 20AA + medium at the same concentration as in cellular uptake study. Similar, these NPs were also individually mixed with 20AA+ medium at the same concentration as controls. 100 µL of the mixture was added into each well of a heparan sulfate (HS) coated 96-well plate (Bio-world). The plate was then incubated in a cell incubator at 37 °C for 1 h. After incubation, the wells were washed by DPBS for three times, then 100 µL of 20AA+ medium was added back into each well, followed by the addition of 20 µL of etchant and 20 µL 0.2 M sodium ascorbic. A plate without washing was added with the etchant and sodium ascorbic similarly. The fluorescence of each well was then measured by a microplate reader (FlexStation 3, Molecular Devices. CA, US).

### The bystander uptake inhibition

For HS inhibition, CHO and H1975 cells were incubated with 10 µg/mL HS (H4784, Sigma) 10 min prior the addition of individual bystander NPs (Ag-647, Au-647, IONP-647 or QD-647) plus 10 µM D-TAT-NH_2_ or T-Ag in DMEM medium. 1 h after incubation of NPs, cells were etched and analyzed by flow cytometry. CHO^pgs745^ cells were treated similarly for NP incubation but without HS treatment, and analyzed by flow cytometry. For macropinocytosis inhibitors, CHO and H1975 cells were received 1 h pre-incubation with 50 µM cytochalasin D, 2.5 mM MβCD, 10 µM EIPA, or 1 µM rottlerin in DMEM, followed by the incubation with Ag-647 and T-Ag, or with T-Ag alone for another 1 h. Cells were then treated as above and analyzed by flow cytometry. For Cys inhibitors, CHO and H1975 cells were pre-incubated with 100 µM erastin (Cat# 5449, TOCRIS) or 2 mM glutamate for 10 min, then incubated with T-Ag and Ag-647 in the presence of corresponding inhibitors (erastin 100 µM, glutamate 2 mM) for 30 min at 37 °C. The cells were then etched and analyzed using flow cytometry.

### In vitro imaging

CHO cells were maintained in growth media (F-12K + 10% FBS and Penicillin/ Streptomycin) on glass chamber slides for 24 h (eight chamber, Nunc). The cells were then washed with DPBS once and incubated with Ag-488 or QD-488, with or without T-Ag in DMEM medium for 1 h at 37 °C. Here, 2x concentration of NPs was used compare to flow cytometry study. After incubation, cells were etched for 1 min, aspirated, then washed with DPBS twice and fixed with 4% PFA. The chamber slides were then mounted in DAPI-containing mounting medium (Vector Laboratories, Burlingame, CA) with a coverslip and examined under a Zeiss LSM 710 NLO confocal microscope. Cells were also incubated in AA-free and Cys medium mixed with Ag-488 and T-Ag and treated similarly to image.

### Transmission electron microscopy

All NPs were examined using TEM (JEOL 1200 EX II TEM) for their core size. CHO cells were incubated with Au50 or T-Au + Au 50 in DMEM medium for 1 h, then processed and imaged by with JEOL 1200 EX II TEM (JEOL) as previously described^18^.

### Ex vivo tissue uptake

All animal work was approved by the Institutional Animal Care Committee of the Sanford Burnham Prebys Medical Discovery Institute. Live 4T1 tumors slices were sliced with a Leica VT1200S vibratome at a thickness of ~200 µm as previously reported^18^. The slices were cultured at the liquid–air interface of 12-well cell culture insert (Cat 353103, BD Biosciences) in DMEM medium with 10% dialyzed FBS for 4 h at 37 °C. The tumor slices were then washed with pre-warmed PBS for 3 times, and incubated with Ag-488 in 20AA+, or with Ag-488 and T-Ag in AA-free, 20AA+, and Cys medium for 2 h at 37 °C. After incubation, the slices were washed with PBS, exposed to etchant in PBS for 1 min, washed, fixed with 4% PFA, and mounted with the DAPI-containing mounting medium. Zeiss LSM 710 NLO confocal microscope was used to examine the tumor slices. For the bystander uptake of macromolecules, FITC-dextran was used as cargo probes. The 200 µm tumor slices were incubated in FBS free DMEM medium for 4 h at 37 °C^43^, then washed with pre-warmed PBS for three times, and incubated with 0.2 mg/mL FITC-dextran in FBS free 20AA+, or with FITC-dextran and T-Ag in AA-free, 20AA+, and Cys medium (FBS free medium) for 1 h. After incubation, the tumor slices were treated as above and imaged by confocal microscope.

### In vivo bystander uptake

For in vivo bystander uptake of AgNPs, 4T1 tumor bearing mice were intratumorally (i.t.) injected with 20 µL of OD (optical density) 200 Ag-555, T-Ag, or T-Ag plus 0.4 mM Cys respectively. 10 min after i.t. injection, all mice were intravenously (i.v.) injected with 100 µL of OD 40 Ag-647 in PBST (1x PBS with 0.005% Tween 20). For in vivo bystander uptake of macromolecules, 4T1 tumor bearing mice were i.t. injected with 20 µL of OD 200 Ag-555, or T-Ag with 0.4 mM Cys for 10 min prior to the i.v injection of FITC-dextran (0.2 mg FITC-dextran in 100 µL PBS). 2 h after i.v. injection, mice were transcardial perfused with PBS and then tumors were collected and further dissociated to single cells using MACS tumor dissociation kit (Miltenyi Biotec, Inc. Bergisch Gladbach, Germany). The single cells were then etched, washed with PBS, fixed and analyzed by flow cytometry. The flow cytometry data were analyzed using FlowJo.

### Statistical analysis

All data are represented as mean ± s.d. Each experiment was repeated at least three times, unless otherwise indicated. Statistical analysis was performed using a one-way ANOVA with Tukey’s multiple comparisons test and Graphpad prism was used as statistical software. Calculated *P* values were considered to be significant for *P* < 0.05.

## Supplementary information

supplementary information

source data

## Data Availability

The source data for all figures and supplementary figures in this paper are provided as Source Data files.
