# Supplementary material for: Cellular internalization of bystander nanomaterial induced by TAT-nanoparticles and regulated by extracellular cysteine
Source: Nat Commun. 2019 Aug 13;10:3646. doi: 10.1038/s41467-019-11631-w (PMC6692393; doi:10.1038/s41467-019-11631-w)
Supplement: Supplementary file 1 — supplementary information [file 41467_2019_11631_MOESM1_ESM.pdf]

Supplementary Information for

Cellular internalization of bystander nanomaterials induced by co-administered cationic TAT-nanoparticles and regulated by extracellular Cysteine

Yushuang Wei[1,2], Tang Tang[1,2], Hong-Bo Pang[1,2]*

[1] Department of Pharmaceutics, University of Minnesota, Minneapolis, MN

[2] Cancer Center, Sanford Burnham Prebys Medical Discovery Institute, La Jolla, CA

*Correspondence: pang0051@umn.edu

Supplementary Methods

Silver nanoparticles (AgNPs)

Peptide-coated AgNPs and neutravidin (NA) complexes were assembled through biotin-neutravidin interaction [1, 2]. Generally, 30 nm AgNPs were synthesized via the reaction of $AgNO_3$ and sodium citrate and then coated with HS-PEG1000-NH_2 and OPSS-NA (NA pre-coated with OPSS-PEG5000-NHS through NHS-amine reaction) to get NA-Ag-NH_2 complexes (optical density (OD) 300). The complexes can be easily labeled by reacting with NHS-CF488A, NHS-CF555, NHS-CF647 dyes to obtain CF488-Ag-NA, CF555-Ag-NA and CF647-Ag-NA. By mixing with biotin DMSO solution, the NA binding sites were blocked by biotin to get the bystander or control AgNPs: CF488-Ag-NAB (Ag-488), CF555-Ag-NAB (Ag-555) and CF647-Ag-NAB (Ag-647). For TAT peptide conjugation, biotin-D-TAT-NH_2 peptides (aqueous) were mixed with biotin DMSO solution at molar ratio 1: 4, and then added to CF555-Ag-NA solution, reacted at room temperature for 30 min. The mixture was then centrifuged at 7200 g, 10 min to remove free biotin and peptide. After washed with PBST (1x PBS with 0.005% Tween 20) for 3 times, the final CF555-Ag-TAT (T-Ag) was resuspended in PBST at OD 200 (extinction 1.45×10^{10} M^{-1} cm^{-1}, that's about 13.8 nM) [3], which are same to all other AgNPs.

Gold nanoparticles (AuNPs)

Peptide-coated AuNPs and NA complexes were assembled through biotin-NA interaction [1, 2]. Similar to AgNPs, 20 nm AuNPs were synthesized through the reaction of $HAuCl_4$ and sodium citrate and then coated with HS-PEG1000-NH_2 and OPSS-NA. The AuNPs were labeled with CF647, and bound with biotin as the bystander or control CF647-Au-NAB (Au-647). Besides, TAT-Au-647 (T-Au) was obtained by mixing CF647-Au-NA with biotin-D-TAT-NH_2 and biotin mixture. The final concentration of all AuNPs was adjusted to OD 30 in PBST (extinction 5.41×10^8 M^{-1} cm^{-1}, that's 39.5 nM) [4].

Iron oxide nanoparticles (IONPs)

IONPs were synthesized according to a previous report [5]. First, to reduce dextran, 10 g dextran (Mw ~10,000 Da) dissolved in 100 mL DI water was mixed with 1 g sodium borohydride and stirred overnight at room temperature. The pH of the solution was adjusted to 6.0 using 6 M HCl. The mixture was then dialyzed (molecular weight cut-off, MWCO 3500 Da) against DI water for 48 h, followed by lyophilizing to produce a white solid reduced dextran. To synthesize IONPs, 400 mg reduced dextran and 292 mg $FeCl_3 \cdot 6H_2O$ were dissolved in 18 mL degassed DI water and stirred in an ice-water bath for 30 min with nitrogen bubbling. Then a solution of 143 mg $FeCl_2 \cdot 4H_2O$ in 4 mL degassed DI water was prepared and added into the mixture. 5 minutes after the addition of $FeCl_2$ solution, 670 µL of ice-cold $NH_4 \cdot OH$ was dropped into the flask with a syringe under rapid stirring. The solution was then heated to 85 °C and kept at 85 ± 5 °C for 2 h. After cooled down to room temperature, the solution was dialyzed (MWCO 50 kDa) for 72 h against DI water. Finally, the solution was collected, concentrated by 50 kDa (nominal molecular weight limit, NMWL) centrifugal filter (4000 g, 20 min), and lyophilized to obtain a brown solid.

The IONPs were then aminated and conjugated with TAT peptide using a modified protocol as previous described[6]. To crosslink the nanoparticles, IONPs were dissolved in DI water at 10 mg Fe/mL. 3 mL IONPs colloid was added to 5 mL NaOH (5 M) and 2 mL epichlorohydrin and then agitated for 24 h at room temperature. After reaction, the mixture was dialyzed against double distilled water for 24 h by using a dialysis cassette (MWCO 3500 Da). The solution was collected and amination was done by adding 0.05 mL of concentrated $NH_4 \cdot OH$ (30%) to 1 mL of crosslinked IONPs colloid (~10 mg Fe/mL). The mixture was agitated at room temperature for 48 h and then dialyzed (MWCO 3500 Da) against double distilled water for 24 h. After dialysis, The IONP-NH_2 solution can be further purified and concentrated using 50 kDa centrifugal filters (4000 g, 20 min). The concentration of amine group on IONPs can be determined by o-Phthalaldehyde reaction according to the instruction (FT-02727A,

Interchim). IONP-NH_2 could be easily labeled by reacting with NHS-CF647 dyes to obtain IONP-647 as the bystander NPs. To conjugate TAT peptide, IONP-NH_2 in PBS was reacted with NHS-PEG4-DBCO to make IONP-DBCO. Meanwhile, NA solution was mixed with NHS-PEG5000-Azide to obtain Azide-NA. The reaction residue was removed by 30 kDa centrifugal filters (4000 g, 15 min). IONP-NA complex was then made by mixing IONP-DBCO and Azide-NA solution and reacted at 4°C overnight. The product was purified by 100 kDa centrifugal filters (4000 g, 20 min). The obtained IONP-NA was labeled with NHS-CF647 dye through the rest free amine groups, and then mixed with biotin-D-TAT-NH_2 peptide solution and reacted at room temperature for 30 min to finally get the D-TAT-IONP-CF647 (T-IONP). The final concentration of Fe in T-IONP (Same as IONP-647) solution was adjusted to 1 mg Fe/mL with PBS.

QD (ZnS)

The synthesis of ZnS QD was adapted from a previous QD protocol [7]. 110 mg zinc acetate was dissolved in 5 mL DI water for a final concentration of 0.1 M. 298 mg 3-Mercaptopropionic acid (3-MPA) was diluted in 20 mL water. The two solutions were mixed together and brought up to a total volume of 35 mL using H_2O. The pH was adjusted to 10.3 ± 0.2 with 2 M NaOH aqueous solution. It was then degassed with N_2 for 30 min. 108 mg Na_2S was dissolved in 4.5 mL DI H_2O (0.1 M), added to the degassed solution, and quickly mixed well. The final solution was heated up to 50 °C for 2.5 h and cooled to room temperature. 80 mL of ethanol was added to the solution to precipitate QD. The suspension was spun at 1000 g for 3 min and the supernatant was removed. The precipitate was washed two more times with ethanol and spun to remove residual MPA and metal ions. The white precipitate was dissolved in DI H_2O to be used directly for characterization or lyophized for storage. For QD amination, 4 mg QD powder was dissolved in 0.5 mL PBS and mixed with HS-PEG2000-NH_2 (40 mg, in 1 mL PBS). The solution was incubated at 50 °C overnight and purified using 10 kDa centrifugation filter (3000 g, 10 min) to remove the free PEG molecules. The concentration of amino group on QD-NH_2 was measured by o-Phthalaldehyde reaction as above. QD-NH_2 then can react with NHS-CF488A or NHS-CF647 dyes to make QD-488 or QD-647 as the bystander NPs.

The conjugation of TAT peptide on QD-NH_2 was similar to IONP-NH_2. NHS-PEG4-DBCO was used as the crosslinker to link azide-NA and QD-NH_2 to obtain QD-NA. Biotin-D-TAT-NH_2 peptides (aqueous) were mixed with biotin DMSO solution at molar ratio 1: 4, and then mixed with CF647 labeled QD-NA at room temperature for 30 min. D-TAT-QD-CF647 (T-QD) was then purified by 10 kDa centrifugal filters (3000 g, 10 min). The final concentration of T-QD (Same as QD-488 and QD-647) was adjusted to 0.5 mg/mL with PBS.

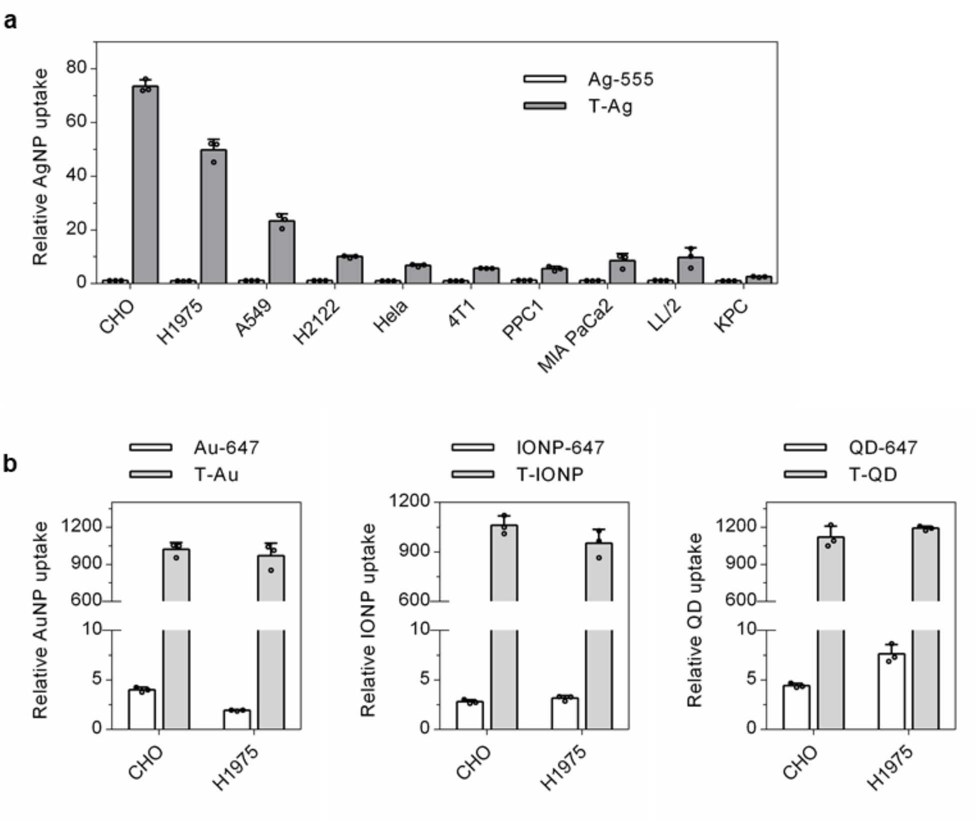

Supplementary Fig. 1. **Cellular uptake of T-NPs**.

a, T-Ag uptake in various types of cells. T-Ag-555 and Ag-555 were incubated with indicated cells in DMEM medium for 1h, etched and analyzed by flow cytometry. The quantity of internalized T-Ag as fluorescence intensity per cell was normalized to that of corresponding cells and plotted on the y axis. Data shown here are mean ± standard deviation (s.d.) of three independent experiments (n = 3). CHO and H1975 cells showed higher T-Ag uptake than other cells.

b, The uptake of T-Au, T-IONP and T-QD in CHO and H1975 cells. Indicated NPs were incubated with CHO and H1975 cells in DMEM medium for 1 h and quantified as described in **a**. The quantity of internalized NPs as fluorescence intensity per cell was normalized to that of cells only and plotted on the y axis. Data presented here are mean ± s.d. of three independent experiments (n = 3). Source data are provided as a Source Data file.

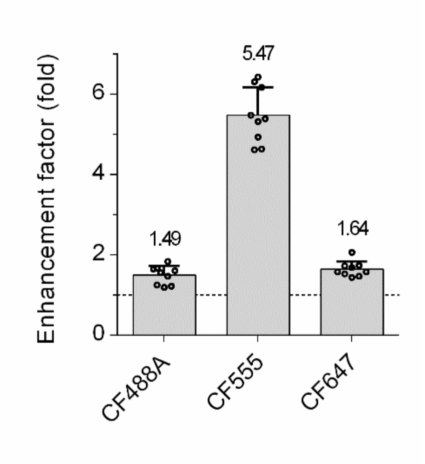

Supplementary Fig. 2. Enhancement factors of AgNPs

AgNP-NA loaded with CF488A, CF555 and CF647 (Ag-488, Ag-555, Ag-647) were synthesized as described above. The enhancement factors (EFs) were determined according to a previously reported protocol [1]. Fluorescence intensity of AgNP-Dye before and after etching was measured and used to calculate EFs. Basically, 100 µL of PBS was added into columns 2-6 of a 96 well plate. For column 1, 130 µL of PBS or etchant was filled. Then 20 µL of OD 200 AgNP-dye or control PBS was added to column 1 and mixed uniformly. One minute later, 50 µL of the mixture was transferred to column 2, mixed evenly, then 50 µL was transferred to column 3, and so on for column 4~6 to a 3-fold dilution series. In column 6, 50 µL of solution was removed to keep all wells having the same volume (100 µL). Fluorescence intensity of each well was measure by a microplate reader. Control PBS were used as background to be subtracted form respective AgNP-dye. The fluorescence intensity ratio of non-etched to etched for each dye was calculated. The highest and lowest (less than 3 times of background signal) concentrations were not used in subsequent analysis and the middle concentration ratios were averaged. Triplicate experiments were performed and used to calculate the average EFs. Source data are provided as a Source Data file.

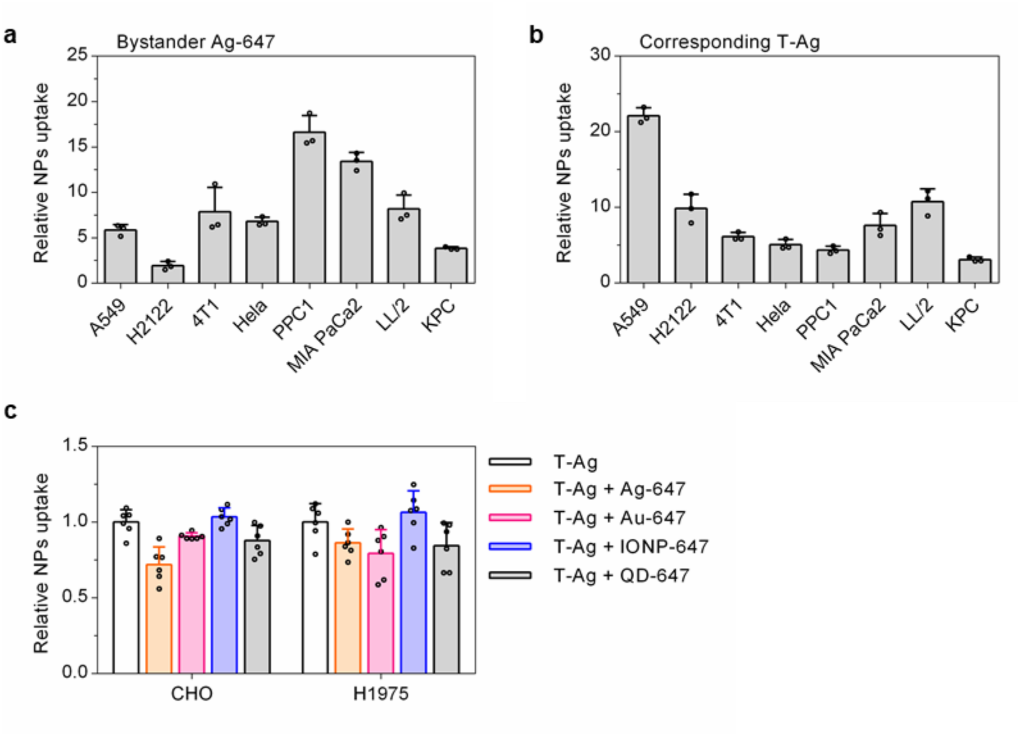

Supplementary Fig. 3. Validation of T-NP-induced bystander uptake.

a-b, T-Ag-induced bystander uptake in indicated cells. **a**, bystander uptake of Ag-647; **b**, corresponding T-Ag uptake. The indicated cells were incubated in DMEM medium with T-Ag and 647-Ag for 1 h. After etching and washing, the fluorescence intensity of internalized NPs per cell was determined by flow cytometry and normalized to that of untargeted NPs (Ag-647 in **a**, Ag-555 in **b**) alone in corresponding cells (y-axis). Data presented here are mean ± s.d. of three independent experiments (n = 3).

c, T-Ag uptake when incubated together with Ag-647, Au-647, IONP-647 and QD-647 (Refer to Fig. 1a). T-Ag uptake in each group was normalized to the internalization of T-Ag alone. Data are expressed as mean ± s.d. of three independent experiments (n = 6). Source data are provided as a Source Data file.

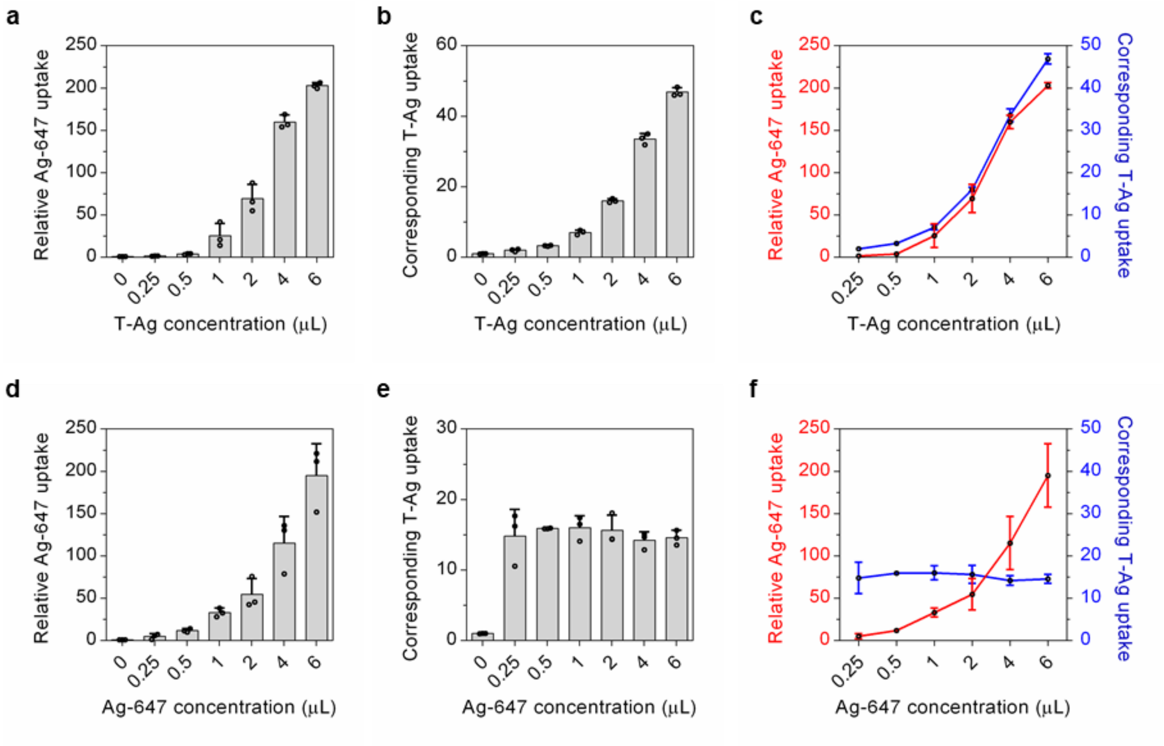

Supplementary Fig. 4. Increasing T-Ag or bystander Ag-647 dosage enhanced bystander NP uptake.

a-c. Cellular internalization of bystander Ag-647 and corresponding T-Ag increased at higher T-Ag concentrations. Two microliters of Ag-647 were mixed with 0.25, 0.5, 1, 2, 4 or 6 µL of T-Ag in 100 µL DMEM medium separately and then incubated with H1975 cells for 1 h. After incubation, cells were etched and the fluorescence intensity of internalized NPs per cell was quantified by flow cytometry and normalized to that of cells only. Data presented here are mean ± s.d. of three independent experiments (n = 3). **a**, relative Ag-647 uptake; **b**, corresponding T-Ag uptake; **c**, comprehensive diagram of **a** and **b**.

d - f. The bystander uptake of Ag-647 increased as the amount of Ag-647 increased. Similar to **a-c**, 2 µL of T-Ag were mixed with 0.25, 0.5, 1, 2, 4 or 6 µL of Ag-647 in 100 µL DMEM medium and then incubated with H1975 cells for 1 h. The fluorescence intensity of NPs per cell was quantified as in **a** - **c**. Data presented here are mean ± s.d. of three independent experiments (n = 3). **d**, relative Ag-647 uptake; **e**, corresponding T-Ag uptake; **f**, comprehensive diagram of **d** and **e**. The bystander uptake of Ag-647 increased as the amount of Ag-647, while the corresponding T-Ag uptake remained similarly. Source data are provided as a Source Data file.

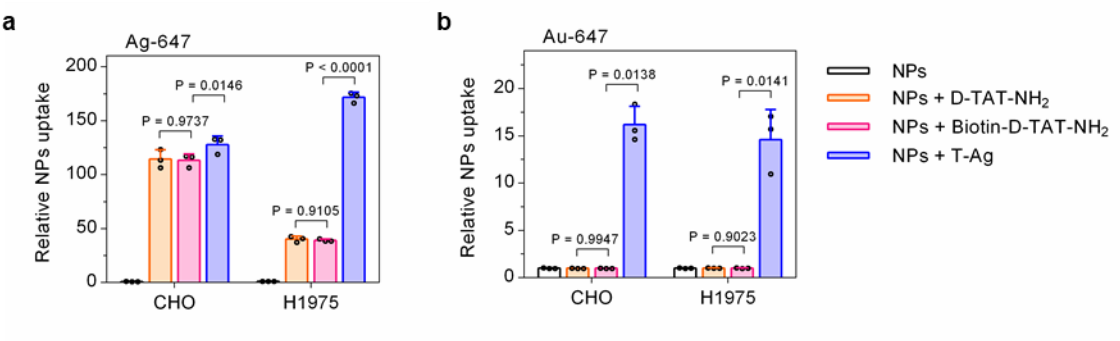

Supplementary Fig. 5. Bystander uptake induced by biotin-TAT peptide after biotin blockage.

a, Bystander uptake of Ag-647 induced by Biotin-TAT peptide. Cells were incubated in DMEM medium with Ag-647 plus 10 μM biotin-D-TAT-NH_2, or 10 μM D-TAT-NH_2 or T-Ag for 1 h, etched and then analyzed by flow cytometry. The quantity of internalized Ag-647 as fluorescence intensity per cell was normalized to that of Ag-647 alone (y-axis).

b, Bystander uptake of Au-647 induced by Biotin-TAT peptide. The cells were treated similarly as in **a**, but the bystander NPs was changed to Au-647. The quantity of internalized Au-647 as fluorescence intensity per cell was normalized to that of Au-647 alone (y-axis). Data were analyzed using one-way ANOVA with Tukey's multiple comparisons test and are shown as mean ± s.d. of three independent experiments (n = 3 for a and b). One-way ANOVA for c, CHO, F = 564.68, P < 0.0001. c, H1975, F = 3107.6, P < 0.0001; d, CHO, F = 180.88, P = 0.0055. d, H1975, F = 178.69, P = 0.0056. Source data are provided as a Source Data file.

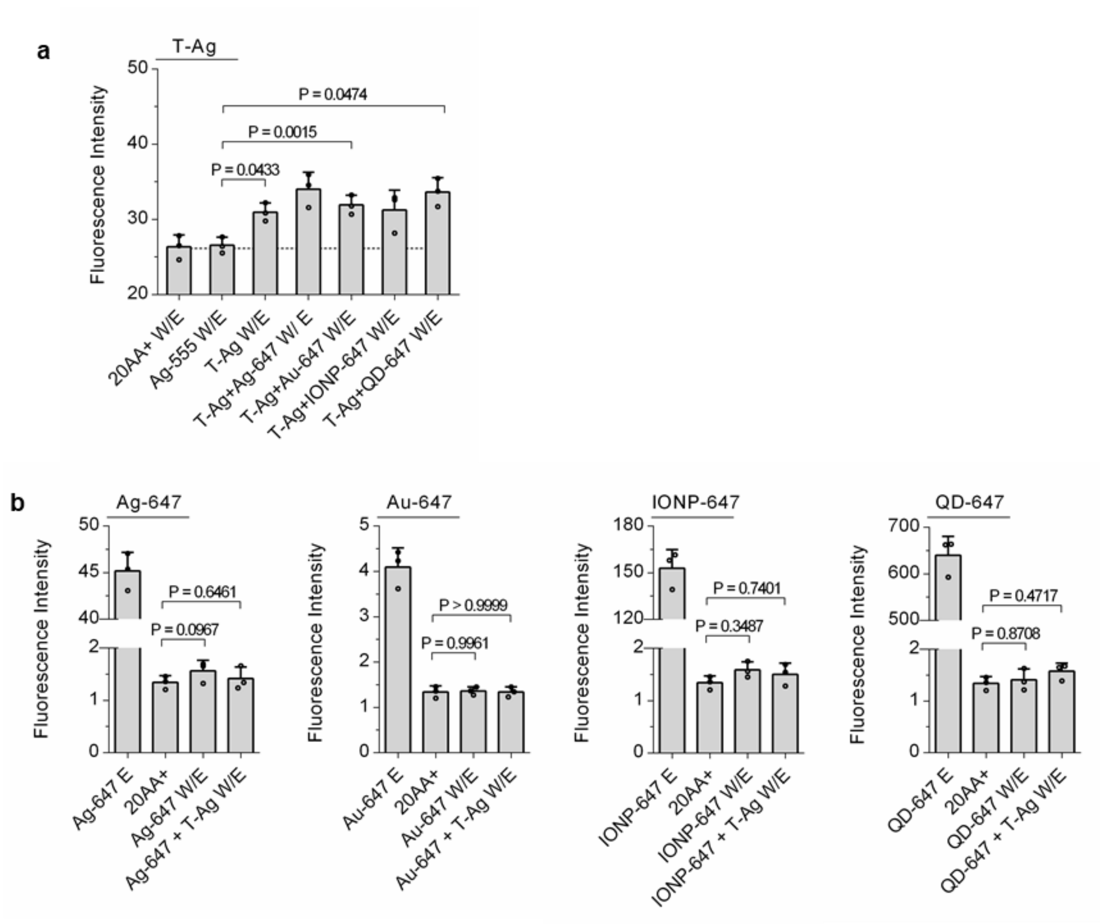

Supplementary Fig. 6. The bystander NPs were not physically attached to T-NPs in plate binding assay

a, Fluorescence signal of T-Ag bound to heparan sulfate coated plate. T-Ag was mixed with Ag-647, Au-647, IONP-647 or QD-647 in 20AA+ medium at cell treatment concentration and incubated in the heparan sulfate (HS) coated 96-well plate at 37 ˚C for 1 h. T-Ag alone and Ag-555 alone were also included as control. After incubation, NPs in solution was aspirated. Each well was washed with PBS for 3 times, and 20AA+ medium was added back, followed by the addition of etchant and sodium ascorbic solution (washing and etching, W/E). The CF555 signal of T-Ag in each well was measured using a plate reader with the setting: excitation 532 nm, cut-off 550 nm, emission 565 nm. Data were analyzed using One-way ANOVA with Tukey's multiple comparisons test and are shown as mean ± s.d. of three independent experiments (n = 3). One-way ANOVA, F = 26.964, P = 0.0234.

b, No bystander NPs bound to T-Ag. Bystander NPs (Ag-647, Au-647, IONP-647 or QD-647) alone or with T-Ag were incubated similarly as in **a**, and treated with only etchant for the original signal (E), or washed and etched (W/E) for bound signal (on plate and T-Ag). The CF647 signal of bystander NPs in each well was measured with the following setting: excitation 628 nm, cut-off 630 nm, emission 665 nm. Data were analyzed using One-way ANOVA with Tukey's multiple comparisons test and presented are mean ± s.d. of three independent experiments (n = 3). One-way ANOVA, for Ag-647, F = 1202.9, P = 0.0008; for Au-647, F = 89.943, P = 0.0080; for IONP-647, F = 486.54, P = 0.0020; for QD-647, F = 749.65, P = 0.0013. Source data are provided as a Source Data file.

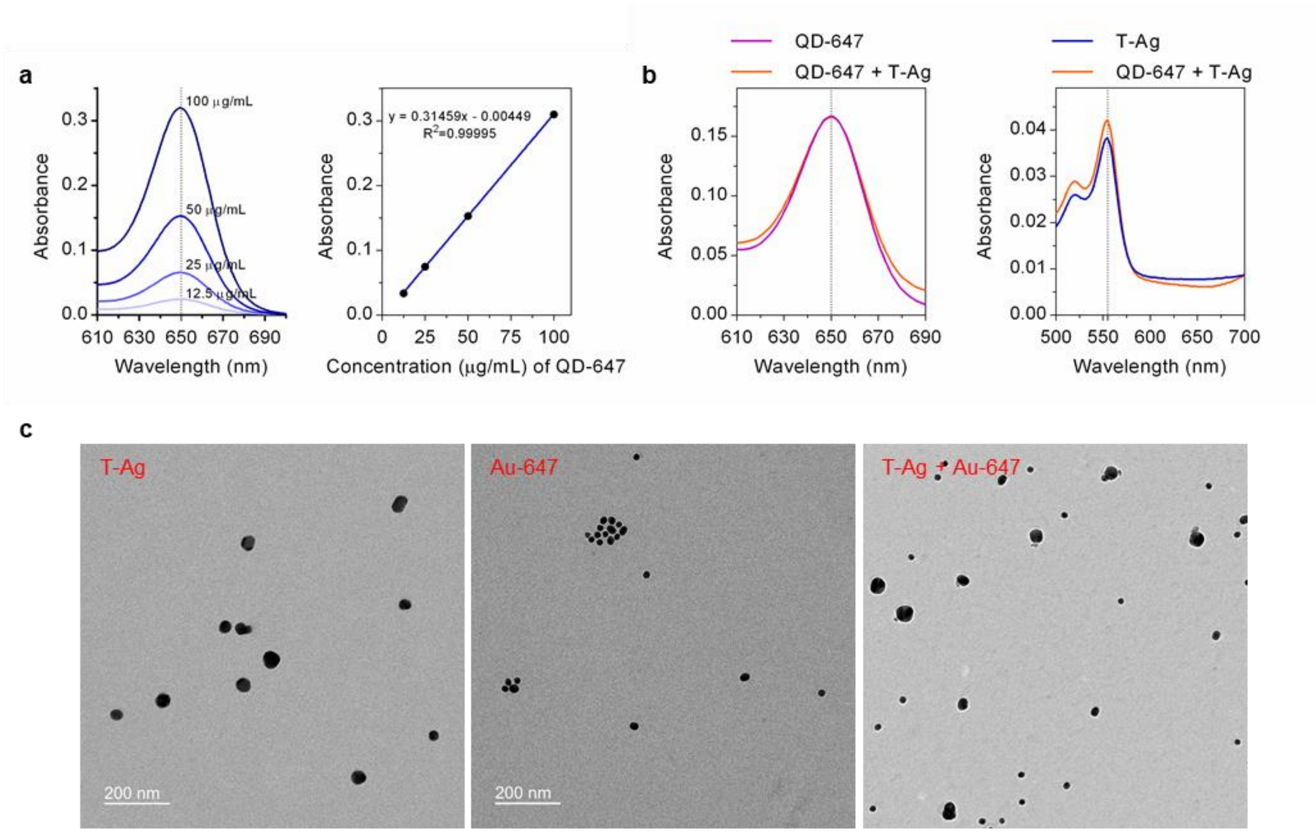

Supplementary Fig. 7. T-NPs did not show physical interaction with bystander NPs in additional assays

a-**b**, No QDs precipitated with T-Ag. QD-647 and T-Ag were used to test the physical interaction by their difference in density: T-Ag can be spun down under 7200 x g while QD-647 remains in the supernatant even at 10, 000 x g. The linear concentration range of QD-647 was firstly determined by UV-Vis spectra of 12.5, 25, 50 and 100 μg/mL QD-647 in PBS. Then 10 μL of QD-647 (1 mg/mL) was added into 190 μL conditional DMEM medium (DMEM containing 10% dialyzed FBS incubated with H1975 cells for 2 h at 37 °C) with or without T-Ag and incubated at 37 °C for 1 h. T-Ag alone was also incubated in the same condition. After incubation, the solution was centrifuged at 8000 x g for 10 min, and the supernatant was collected, then etched to measure the absorbance. The precipitation of T-Ag alone and T-Ag + QD-647 were also carefully washed and etched for the absorbance measurement. **a**, Absorbance spectrum of QD-647 at gradually diluted concentration (left) and the corresponding linear relationship (right). At experimental concentration range, QD-647 absorbance was proportional to the concentration change. **b**, Absorbance spectra of supernatant (QD-647, left) and precipitation (T-Ag, right). The absorbance of supernatant QD-647 did not change, and no absorbance at 650 nm was observed in precipitation, indicating no QDs precipitated with T-Ag.

c, T-Ag did not show physical binding with Au-647. T-Ag and Au-647 at 5× experimental concentration was dispersed in 20AA+ medium at 37 °C for 1 h before making TEM samples. Representative TEM images of T-Ag, Au-647 and T-Ag + Au-647 were shown. Images showed T-Ag dispersed separately from Au-647 without obvious binding or aggregation. Three independent experiments were performed and representative images are shown here. Scale bars, 200nm. Source data are provided as a Source Data file.

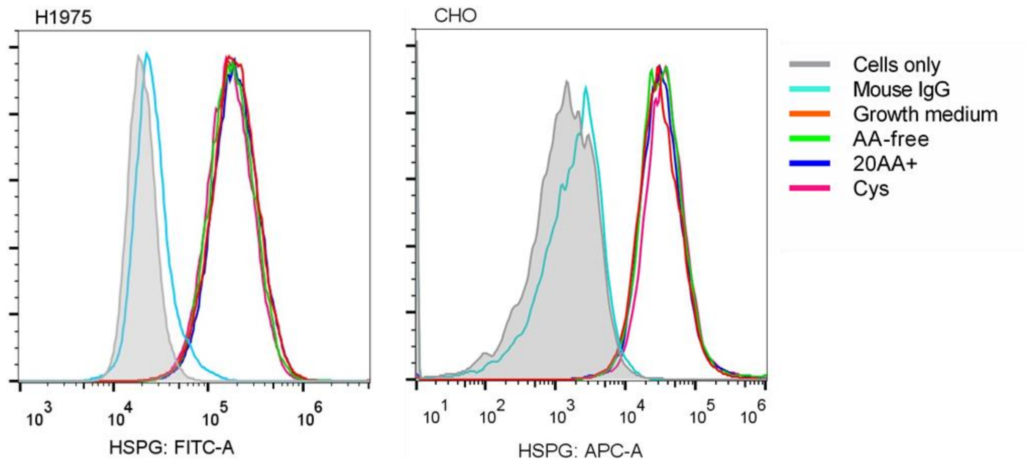

Supplementary Fig. 8. Surface HSPG expression levels in response to media with various AA compositions.

H1975 (left) and CHO (right) cells were incubated in RPMI 1640 (for H1975) or F-12K (for CHO), AA-free, 20AA+ and Cys medium for 1 h at 37 °C, and then incubated with mouse or rat anti-HSPG antibody for 1 h at 4 °C. The cells were then washed with ice-cold PBS and incubated with corresponding 2nd antibodies for another 1 h at 4 °C. After washing with PBS and fixation with 4% PFA, the cells were analyzed with flow cytometry on Novocyte 3000. The flow cytometry data were analyzed using FlowJo. It showed that the surface HSPG level was not affected by different media. Source data are provided as a Source Data file.

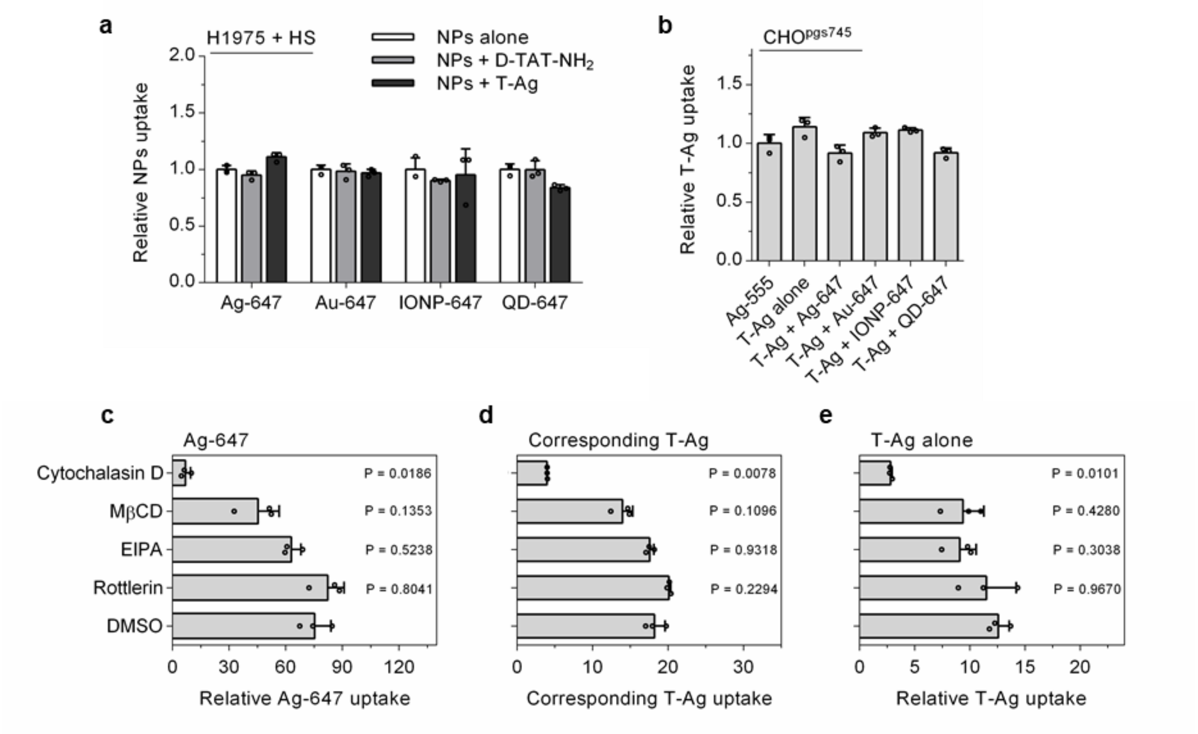

Supplementary Fig. 9. **Mechanistic studies of bystander uptake**.

a, Bystander uptake is completely abolished by HS. Similar to Fig. 2a, H1975 cells were incubated with 10 mg/mL HS prior to the addition of indicated bystander NPs (x-axis) alone, with 10 μM D-TAT-NH_2 or T-Ag. The fluorescence intensity of NPs per cell was quantified by flow cytometry and normalized to that of bystander NPs alone (y-axis). Data presented here are mean ± s.d. of three independent experiments (n = 3).

b, No T-Ag uptake in CHO^{pgs745} cells was observed. CHO^{pgs745} cells were incubated with T-Ag, or with indicated NPs in DMEM medium for 1 h. After etching, the cells were analyzed by flow cytometry. Data shown here are mean ± s.d. of three independent experiments (n = 3).

c-e, Bystander uptake of Ag-647 was inhibited by macropinocytosis (MP) inhibitors. Similar to Fig. 2c-e, H1975 cells were pre-treated with 50 μM cytochalasin D (CD), 2.5 mM methyl-β-cyclodextrin (MβCD), 10 μM ethyl-isopropyl amiloride (EIPA), 1 μM rottlerin or control DMSO for 1 h before incubating with AgNPs. The fluorescence intensity of internalized NPs per cell was quantified by flow cytometry and normalized to that of control AgNPs (Ag-647 for Ag-647 bystander uptake, or Ag-555 for T-Ag uptake) alone. **c**, bystander uptake of Ag-647; **d**, corresponding T-Ag uptake in **c**; **e**, the uptake of T-Ag alone. Data were analyzed using one-way ANOVA with Tukey's multiple comparisons test (inhibitors versus DMSO) and are represented as mean ± s.d. of three independent experiments (n = 3). One-way ANOVA, **c**, F = 88.652, P = 0.0006; **d**, F = 273.98, P < 0.0001; **e**, F = 34.41, P = 0.0211. Source data are provided as a Source Data file.

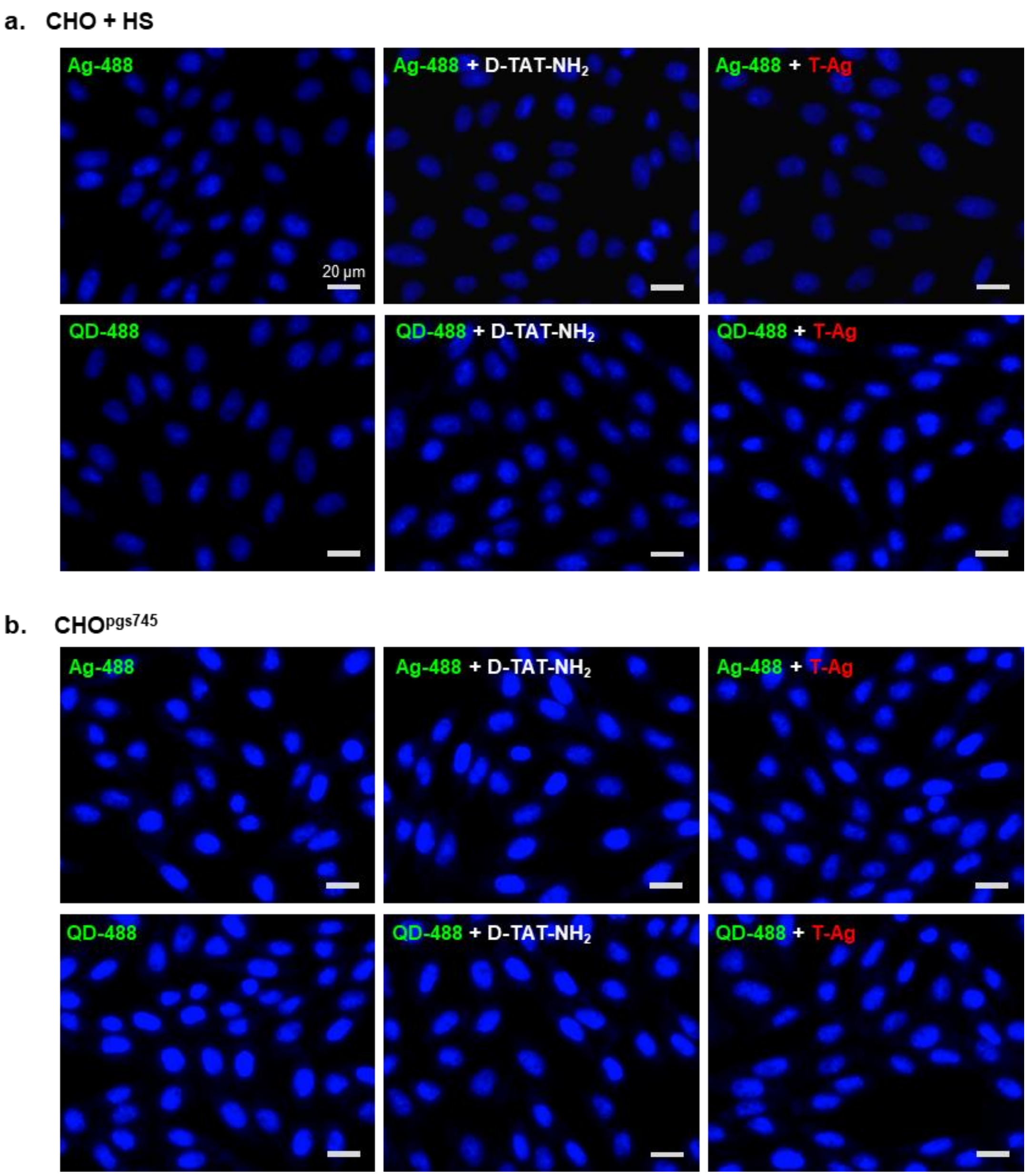

Supplementary Fig. 10. Bystander uptake was completely blocked by HS inhibition and HSPG depletion

a, Bystander uptake were completely abolished by HS. Similar to Fig. 2a, CHO cells were incubated with 10 μg/mL HS 1 h prior to the addition of Ag-488 or QD-488 alone, or with 10 μM D-TAT-NH_2 or T-Ag in DMEM medium and then incubated at 37 °C for another 1 h. After incubation, cells were etched and stained with Hoechst (blue), imaged with an EVOS M5000 imaging system. No bystander uptake, as well as related T-Ag uptake can be seen.

b, CHO^{pgs745} cells did not show bystander uptake. Similar to Fig. 2b, CHO^{pgs745} cells were incubated with Ag-488 or QD-488 alone, plus 10 μM D-TAT-NH_2 or T-Ag in DMEM medium at 37 °C for 1 h. Cells were treated similarly as in **a** and imaged with EVOS M5000. CHO^{pgs745} cells, which do not express HSPG, did not take up bystander NPs and related T-Ag. Three independent experiments (n = 3) were performed and representative images are shown here. Source data are provided as a Source Data file.

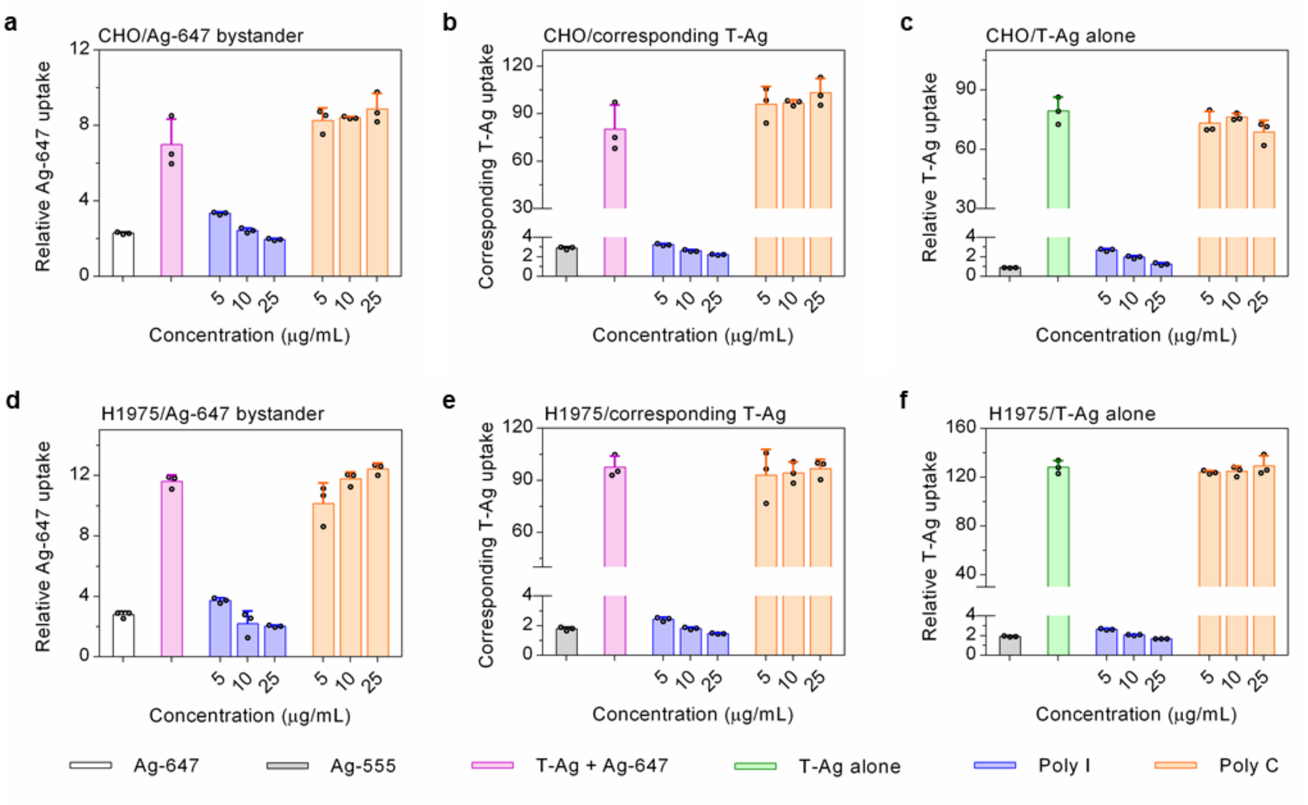

Supplementary Fig. 11. Bystander uptake was blocked by a scavenger receptor inhibitor.

CHO (**a**-**c**) and H1975 (**d**-**f**) cells were pre-incubated with 5, 10, 25 μg/mL polyinosinic acid (Poly I) or polycytidylic acid (Poly C) for 1 h before adding Ag-647 + T-Ag (**a, b**; **d, e**) or T-Ag alone (**c, f**) and incubated for another 1 h. After incubation, cells were etched, detached and then the fluorescence intensity of internalized NPs per cell was quantified by flow cytometry and normalized to that of cells alone. Data presented here are mean ± s.d. of three independent experiments (n = 3). T-Ag uptake, as well as the bystander Ag-647 uptake was completely blocked by poly I, but not the negative control poly C, indicating scavenger receptors were involved in the receptor-mediated MP for both T-Ag and bystander NPs internalization. Source data are provided as a Source Data file.

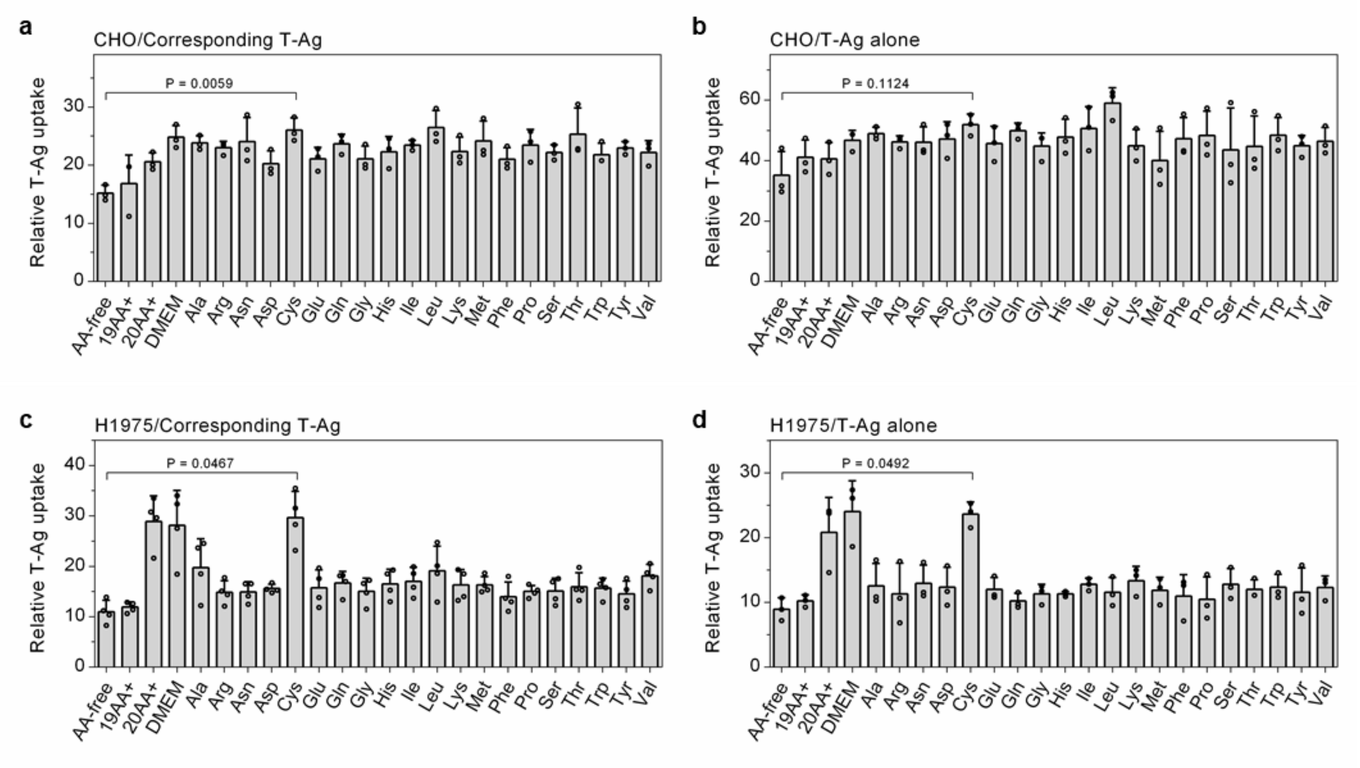

Supplementary Fig. 12. The effect of individual amino acid on T-Ag internalization into cells.

a and **c**, Corresponding T-Ag internalization referring to Fig 4a, b. The internalization of T-Ag in CHO (**a**) and H1975 (**c**) cells as fluorescence intensity per cell was normalized to that of cells alone. Data were analyzed using one-way ANOVA with Tukey's multiple comparisons test and are expressed as mean ± s.d. (n = 3 in **a**, n = 4 in **c**). One-way ANOVA, **a**, F = 5.6157, P = 0.1009; **c**, F = 15.376, P = 0.0021.

b and **d**, The uptake of T-Ag alone in related AA-containing media. CHO (**b**) and H1975 (**d**) cells were treated with T-Ag alone in related media and were quantified as described in **a** and **c**. Data shown are mean ± s.d. of three independent experiments (n = 3). One-way ANOVA, **b**, F = 1.5454, P = 0.3192; **d**, F = 9.5536, P = 0.0397. Source data are provided as a Source Data file.

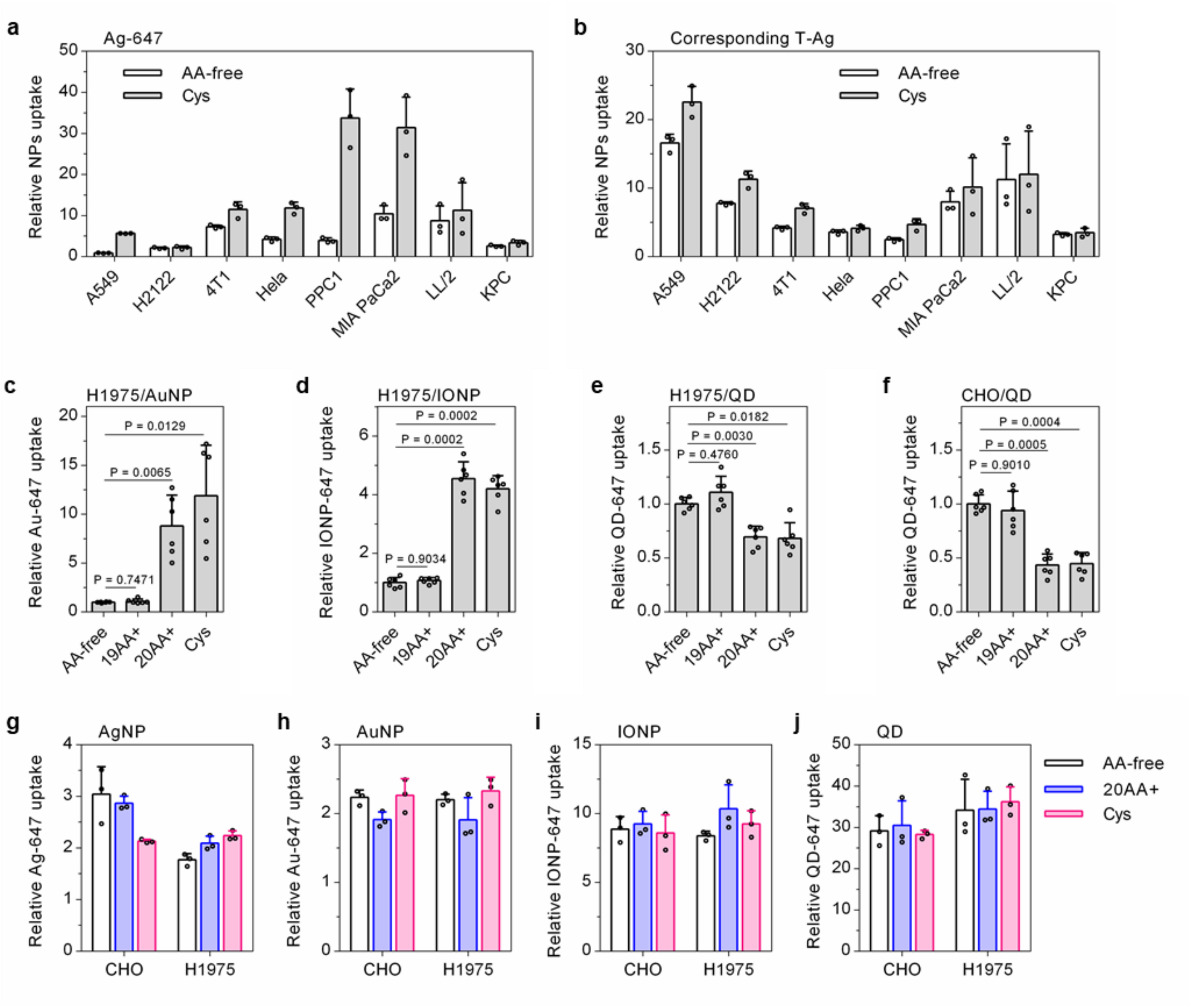

Supplementary Fig. 13. Validation of Cys stimulatory effects on bystander NP uptake

a-b, Cysteine effect on NPs bystander uptake in various cells lines. Indicated cell lines were incubated with Ag-647 and T-Ag in AA-free or Cys medium for 1 h, etched and analyzed by flow cytometry. The internalization of NPs as fluorescence intensity per cell was normalized to that of Ag-647 or Ag-555 (for T-Ag) alone. **a**, bystander uptake of Ag-647; **b**, corresponding T-Ag uptake in **a**. Data shown are mean ± s.d. of three independent experiments (n =3).

c-f, CHO and H1975 cells were incubated in AA-free, 19 AA+, 20AA+ or Cys medium with indicated NPs and T-Ag. After etching and washing, the fluorescence intensity of internalized bystander NPs per cell was determined by flow cytometry and normalized to that in AA-free medium. **c**, Au-647 bystander uptake in H1975; **d**, IONP-647 bystander uptake in H1975; **e**, QD-647 bystander uptake in H1975; **f**, QD-647 bystander uptake in CHO. Data were analyzed using one-way ANOVA with Tukey's multiple comparisons test and are expressed as mean ± s.d. (n = 6). One-Way ANOVA, **c**, F = 28.488, P = 0.0026; **d**, F = 155.29, P < 0.0001; **e**, F = 37.763, P = 0.0002; **f**, F = 56.722, P <0.0001

g-j, Cellular uptake of bystander NPs alone did not show difference in AA-free and Cys medium. CHO and H1975 cells were incubated with Ag-647 (**g**), Au-647 (**h**), IONP-647 (**i**) and QD-647 (**j**) in AA-free, 20AA+ or Cys medium for 1 h and then analyzed by flow cytometry. The internalization of NPs as fluorescence intensity per cell was normalized to that of cells alone. Data shown are mean ± s.d. of three independent experiments (n =3). Source data are provided as a Source Data file.

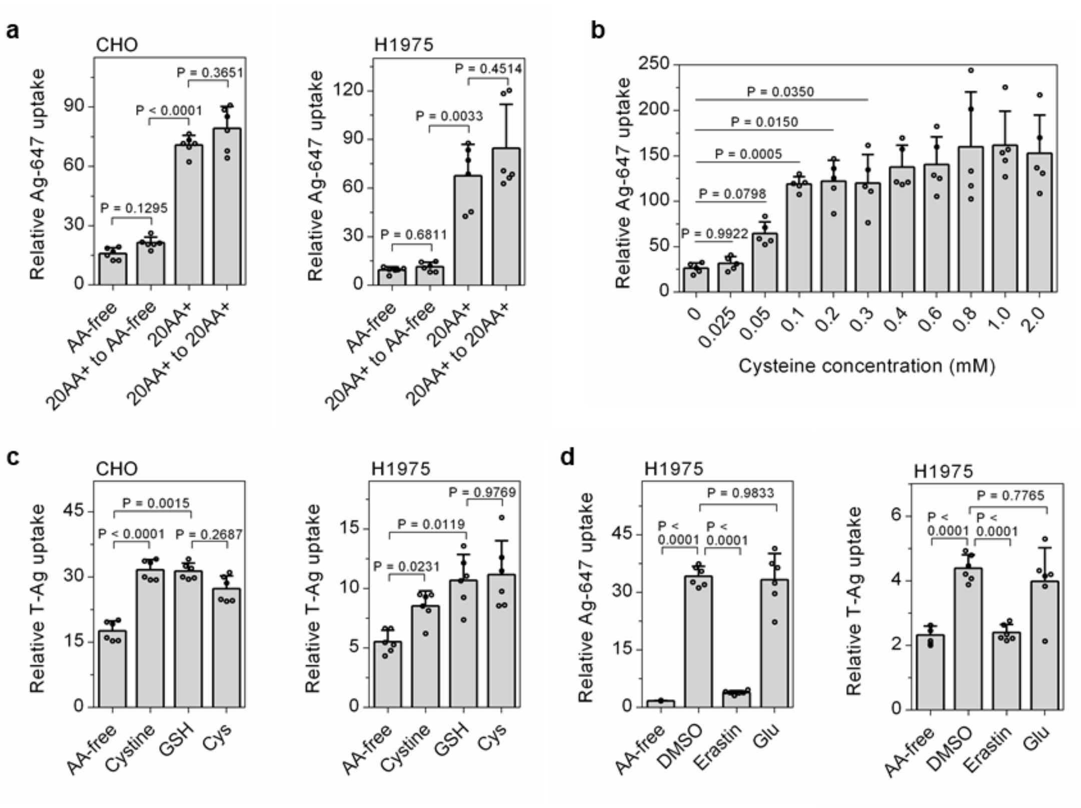

Supplementary Fig. 14. Mechanistic investigation of Cys regulation of bystander uptake.

a, Similar to Fig. 5a, CHO and H1975 cells were pre-incubated in 20AA+ medium for 1 h before exposed to Ag-647 and T-Ag in AA-free or 20AA+ medium for another 1 h. The internalization of Ag-647 as fluorescence intensity per cell was normalized to that of Ag-647 alone. Data are presented as mean ± s.d. of five independent experiments (n = 5). All quantified (**a**-**d**) data were analyzed using one-way ANOVA with Tukey's multiple comparisons. One-way ANOVA, **a** (left), F = 45.287, P = 0.0010; **a** (right), F = 48.590, P = 0.0012.

b, The bystander uptake of Ag-647 increased as Cys concentration increased. Corresponding to Fig 5b, H1975 cells were incubated with Ag-647 and T-Ag in AA-free medium with gradually increasing concentrations of Cys from 0 to 2 mM. After incubation, the cells were etched, and the fluorescence intensity per cell of internalized NPs was determined by flow cytometry, and normalized to that of Ag-647 alone. Data are presented as mean ± s.d. of five independent experiments (n = 5). One-way ANOVA, F = 26.575, P = 0.0010.

c, The T-Ag uptake increased in cystine and GSH medium. Similar to Fig 5c, CHO and H1975 cells were incubated in cystine or GSH-containing medium with T-Ag alone. The fluorescence intensity per cell of internalized NPs was determined by flow cytometry and normalized to that of Ag-555. Data are presented as mean ± s.d. of six independent experiments (n = 6). One-way ANOVA, **c** (CHO), F = 55.091, P = 0.0006; **c** (H1975), F = 12.468, P = 0.0014.

d, Bystander uptake of Ag-647 was inhibited by cystine inhibitors. Similar to Fig 5d, H1975 cells were pre-treated with 100 μM Erastin or 2 mM glutamate for 10 min prior to the incubation with Ag-647 and T-Ag for 30 min in the presence of Erastin (10 μM) or glutamate (2mM). **d** (left), the bystander uptake of Ag-647; **d** (right), the uptake of corresponding T-Ag. The internalized NPs as fluorescence intensity per cell was normalized to that of Ag-647 (For Ag-647 bystander uptake) or Ag-555 (For T-Ag uptake) alone. Data shown are mean ± s.d. of six independent experiments (n = 6). One-way ANOVA, **d** (left), F = 154.48, P < 0.0001; **d** (right), F = 25.697, P = 0.0032. Source data are provided as a Source Data file.

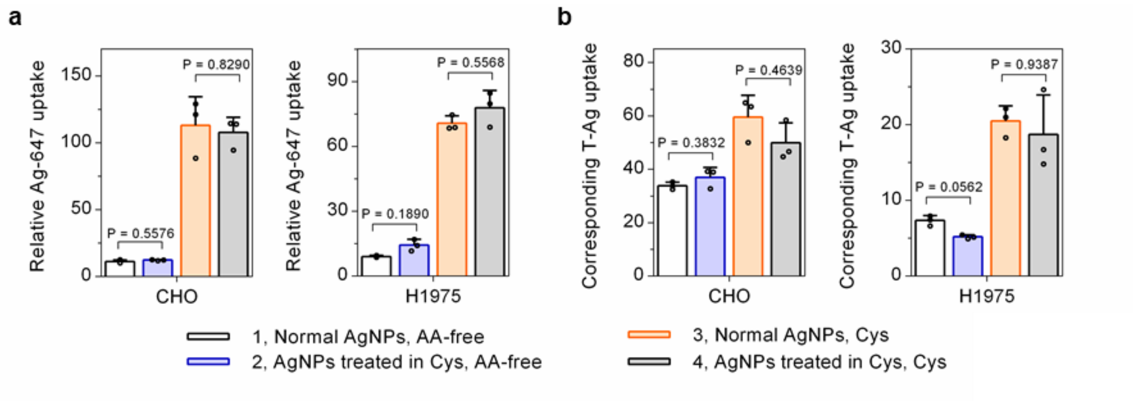

Supplementary Fig. 15. The impact of Cys on the cellular uptake of bystander AgNPs by themselves. T-Ag and 647-Ag were incubated in Cys medium at 37 ˚C for 1 h, followed by washing with PBST and incubation with CHO and H1975 cells in AA-free and Cys medium for 1 h. Cells were also incubated with normal T-Ag and Ag-647 in AA-free and Cys medium. After incubation, cells were etched and then analyzed by flow cytometry. **a**, The bystander uptake of Ag-647; **b**, The corresponding T-Ag uptake for **a**. The internalization of AgNPs as fluorescence intensity per cell was normalized to that of cells alone. Data were analyzed using one-way ANOVA with Tukey's multiple comparisons test and are presented as mean ± s.d. of three independent experiments (n = 3). One-way ANOVA, **a**, for CHO, F = 95.109, P = 0.0095; for H1975, F = 191.03, P = 0.0019; **b**, for CHO, F = 18.671, P = 0.0125. for H1975, F = 23.856, P = 0.0191. Source data are provided as a Source Data file.

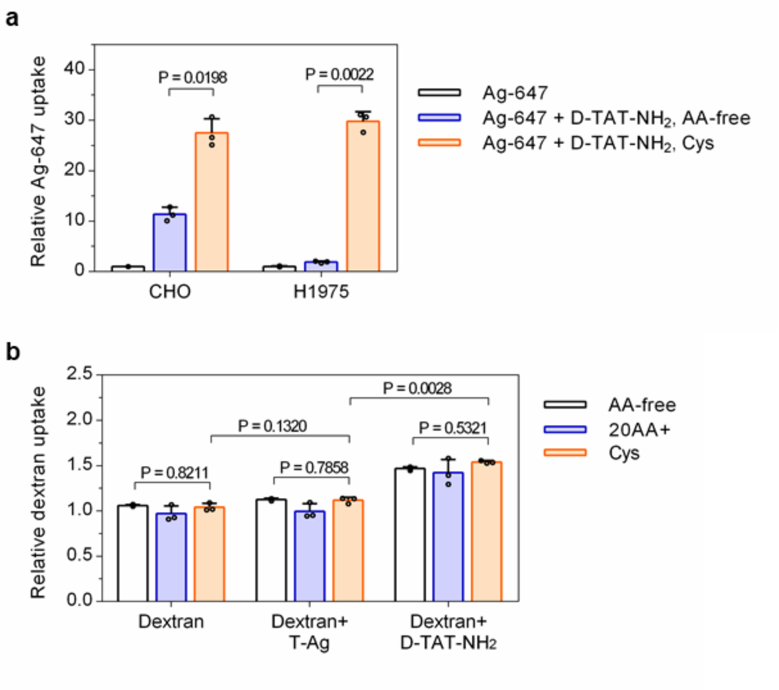

Supplementary Fig. 16. Cys regulation of TAT-peptide-induced bystander uptake and non-NP bystander cargo.

a, Cysteine promoted Ag-647 bystander uptake induced by TAT peptide. CHO and H1975 cells were incubated with Ag-647 and 10 µM D-TAT-NH_2 in AA-free or Cys medium for 1 h. The internalized Ag-647 as fluorescence intensity per cell was normalized to that of Ag-647 alone. Data were analyzed using one-way ANOVA with Tukey's multiple comparisons test and are shown as mean ± s.d. of three independent experiments ($n = 3$). For CHO, one-way ANOVA, $F = 171.83$, $P = 0.0016$. For H1975, one-way ANOVA, $F = 679.10$, $P = 0.0014$.

d, Dextran uptake in AA-free and Cys media. CHO cells were under FBS starvation for 18 h, incubated in AA-free, 20AA+ or Cys medium with 0.2 mg/mL FITC-dextran (70 kDa), or dextran + T-Ag, or dextran + 10 µM D-TAT-NH_2 for 30min, then etched and analyzed by flow cytometry. The internalization of dextran as fluorescence intensity per cell was normalized to that of dextran alone in AA-free medium. Data were analyzed using one-way ANOVA with Tukey's multiple comparisons test and are presented as mean ± s.d. of three independent experiments ($n = 3$). For Dextran, one-way ANOVA, $F = 3.0936$, $P = 0.2198$. For Dextran + T-Ag, one-way ANOVA, $F = 8.0439$, $P = 0.0939$. For Dextran + D-TAT-NH_2, one-way ANOVA, $F = 1.4143$, $P = 0.3536$. Source data are provided as a Source Data file.

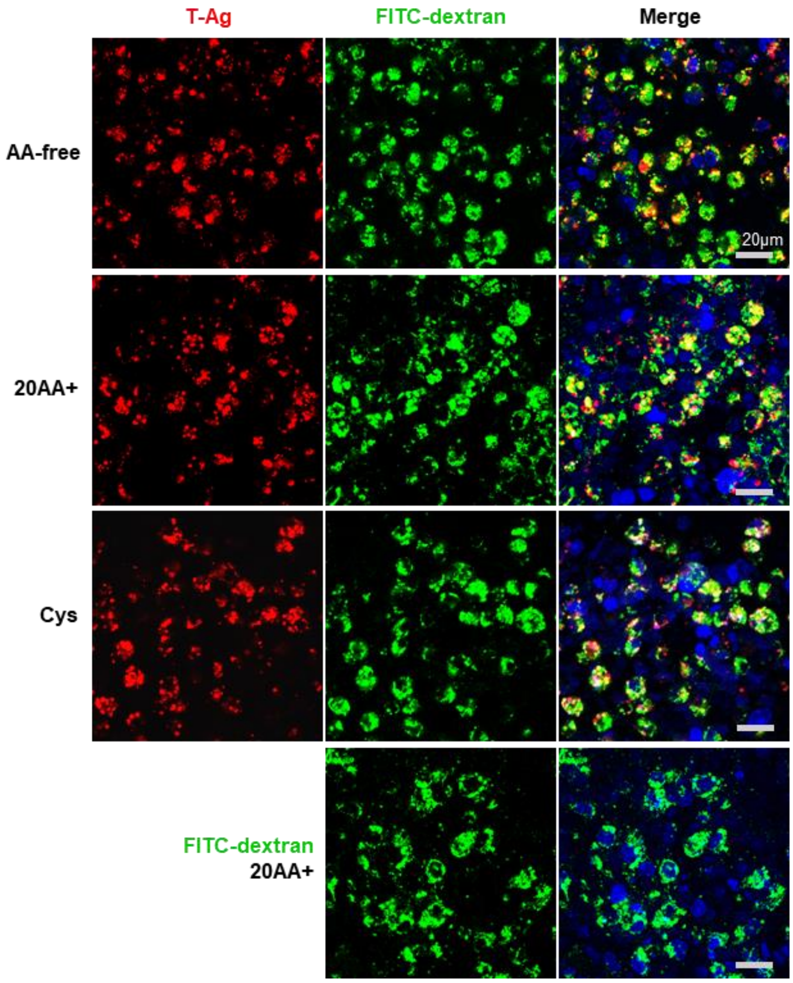

Supplementary Fig. 17. **The uptake of T-NP and dextran in live tumor slices.** T-Ag and FITC-dextran were incubated with live 4T1 tumor slices in AA-free, 20AA+ and Cys medium for 1 h. After incubation, slices were etched, stained with DAPI (blue) and then imaged by confocal microscope (n = 3 mice for each group). Scale bars, 20 μm. Source data are provided as a Source Data file.

Supplementary Table 1. Cells lines used in this study

No.	Cell lines	Types	Species	Note
1	CHO^{WT}	Ovarian	Hamster	HSPG* high expression cells
2	CHO^{pgs745}	Ovarian	Hamster	HSPG non-expression cells
3	A549	Lung	Human	
4	H1975	Lung	Human	
5	H2122	Lung	Human	
6	PPC1	Prostate	Human	
7	LL/2	Lung	Mouse	
8	MIA PaCa2	Pancreatic	Human	
9	KPC	Pancreatic	Mouse	
10	4T1	Breast	Mouse	
11	Hela	Cervical	Human	

*HSPG: Heparan Sulfate Proteoglycans.

Supplementary Table 2. DMEM based amino acid free medium*

No.	Basic reagents	Mw	(1×) mg/L	(1×) mM
1	$CaCl_2$	111	200	1.802
2	$Fe(NO_3)_3 \cdot 9H_2O$	404	0.1	0.000
3	$MgSO_4$	120	97.67	0.814
4	KCl	75	400	5.333
5	$NaHCO_3$	84	3700	44.048
6	NaCl	58	6400	110.345
7	$NaH_2PO_4 \cdot H_2O$	138	125	0.906
8	D-Glucose (Dextrose)	180	4500	25.000
9	HEPES	238	5958	25.034

Supplementary Table 3. Amino acids concentration used to prepare media

No.	Cat#	Amino acids	3-letter	1-letter	Mw	mg/L	mM
A1.	A7627	L-Alanine	Ala	A	89.09	36	0.404
A2.	A5006	L-Arginine	Arg	R	174.2	70	0.399
A3.	A0884	L-Asparagine	Asn	N	132.12	53	0.401
A4.	A9256	L-Aspartic Acid	Asp	D	133.1	53	0.398
A5.	168149	L-Cysteine	Cys	C	121.16	49	0.404
A6.	G1251	L-Glutamic Acid	Glu	E	147.13	59	0.401
A7.	G3126	L-Glutamine	Gln	Q	146.14	584	3.996
A8.	G7126	Glycine	Gly	G	75.07	30	0.399
A9.	H6034	L-Histidine	His	H	155.15	31	0.200
A10.	I2752	L-Isoleucine	Ile	I	131.17	105	0.800
A11.	L8000	L-Leucine	Leu	L	131.17	105	0.800
A12.	L5626	L-Lysine HCl	Lys	K	182.65	146	0.799
A13.	M9625	L-Methionine	Met	M	149.21	30	0.201
A14.	P2126	L-Phenylalanine	Phe	F	164.19	66	0.402
A15.	P0380	L-Proline	Pro	P	115.13	46	0.400
A16.	S4500	L-Serine	Ser	S	105.09	42	0.400
A17.	T8625	L-Threonine	Thr	T	119.12	95	0.798
A18.	T0254	L-Tryptophan	Trp	W	204.23	16	0.078
A19.	T3754	L-Tyrosine	Tyr	Y	181.19	73	0.403
A20.	V0500	L-Valine	Val	V	117.15	94	0.802

* The concentration of all reagents is based on DMEM (SH30022, HyClone) formulation.

Fetal Bovine Serum (FBS) was dialyzed using cassette (MWCO=3,500) into 1X PBS to remove amino acids and glucose. 10% dialyzed FBS was added to the prepared medium and used in this study.

Amino acid free (AA-free) medium was made according to table 3-1. According to table 3-2, individual amino acid was added into AA-free medium respectively to prepare the corresponding 20 individual amino acid additional media (e.g. Ala, Cys, Pro…). All 20 amino acids at corresponding concentration were added into AA-free medium to make the 20 amino acids additional medium (20AA+). Similarly, 19 amino acids, only without Cys were added into AA-free medium to prepare the 19 amino acids additional medium (19AA+).

Supplementary Table 4. DLS size and zeta potential of NPs incubating with Cys

NPs + Cys	Size (nm)	Zeta potential
Ag-647	51.78 ± 4.26	-8.02 ± 0.38
T-Ag-555	57.74 ± 2.53	-5.27 ± 0.81
IONP-647	30.37 ± 2.06	-6.03 ± 1.26
QD-647	19.41 ± 2.19	-8.89 ± 1.56

NPs were incubated with 0.4 mM cysteine in PBS at 37 °C for 1 h and then measured with DLS. Mean ± s.d. N=3. Source data are provided as a Source Data file.

Supplementary References:

1. Braun, G.B. et al. Etchable plasmonic nanoparticle probes to image and quantify cellular internalization. *Nat Mater* **13**, 904-911 (2014).
2. Pang, H.B. et al. An endocytosis pathway initiated through neuropilin-1 and regulated by nutrient availability. *Nat Commun* **5**, 4904 (2014).
3. Paramelle, D. et al. A rapid method to estimate the concentration of citrate capped silver nanoparticles from UV-visible light spectra. *Analyst* **139**, 4855-4861 (2014).
4. Haiss, W., Thanh, N.T., Aveyard, J. & Fernig, D.G. Determination of size and concentration of gold nanoparticles from UV-vis spectra. *Anal Chem* **79**, 4215-4221 (2007).
5. Jarrett, B.R., Frendo, M., Vogan, J. & Louie, A.Y. Size-controlled synthesis of dextran sulfate coated iron oxide nanoparticles for magnetic resonance imaging. *Nanotechnology* **18**, 035603 (2007).
6. Simberg, D. et al. Biomimetic amplification of nanoparticle homing to tumors. *Proceedings of the National Academy of Sciences of the United States of America* **104**, 932-936 (2007).
7. Yin, N.-Q. et al. Preparation and characterization of nontoxic magnetic-luminescent nanoprobe. *Chinese Physics B* **21**, 116101 (2012).
